# Associations between time-series features of training-load monitoring indicators and relative composite performance in elite speed climbers: a GEE and quantile regression study

**DOI:** 10.3389/fphys.2026.1959824

**Published:** 2026-09-11

**Authors:** Shiyi Lu, Shilong Han, Lihan Lin, Qingfu Wang, Wenxin Xu, Guoqing Yuan, Haixu Hu

**Affiliations:** 1 Fujian Normal University, Fuzhou, China; 2 Shanghai University of Sport, Shanghai, China; 3 Mountaineering Administrative Center, General Administration of Sport of China, Beijing, China; 4 Jiangsu Institute of Sports Science, Nanjing, China; 5 China Institute of Sport Science, Beijing, China; 6 Nanjing Sport Institute, Nanjing, China

**Keywords:** athletic performance, generalized estimating equations, quantile regression, speed climbing, time-series features, training-load monitoring

## Abstract

**Objective:**

To examine associations between time-series features of training-load, biochemical, and subjective recovery indicators and relative composite performance in elite speed climbers, and whether these associations differed across conditional performance quantiles.

**Methods:**

Nine elite speed climbers were monitored for 30 weeks, comprising 15 two-week monitoring periods. Six indicators were analyzed: training strain, training monotony, testosterone, creatine kinase (CK), perceived sleep quality, and soreness. Relative composite performance was represented by a sex-specific athlete-level score ranging from 0 to 1. Missing testosterone and CK values were handled using chained-equation imputation with predictive mean matching. Rolling means and coefficients of variation (CVs) were calculated over four consecutive monitoring periods, yielding 12 candidate time-series features and 108 rolling observations. Generalized estimating equations (GEE) assessed population-averaged associations, while athlete-clustered quantile regression explored associations at τ = 0.25, 0.50, and 0.75. False discovery rate correction and sensitivity analyses were applied.

**Results:**

In the primary GEE analysis, CK rolling CV showed a nominal positive association with performance (β = 0.529, 95% CI 0.061–0.997, P = 0.027), but not after FDR correction (FDR = 0.322). In the six-predictor quantile-regression model, training monotony rolling mean was negatively associated with performance across three quantiles, whereas testosterone rolling mean was positively associated and CK rolling mean negatively associated. These directions were relatively consistent in the extended model and sensitivity analyses. Associations involving training strain, perceived sleep quality, and soreness were less consistent.

**Conclusions:**

GEE and quantile regression provided complementary perspectives: GEE characterized population-averaged associations, whereas quantile regression identified differences across the conditional distribution of relative composite performance. Training monotony, testosterone, and CK showed consistent directional patterns. Rolling means and rolling CVs may provide complementary information for interpreting training-load, biochemical, and recovery monitoring in elite speed climbers. These findings require confirmation in samples with repeated objective performance outcomes.

## 1 Introduction

Against the background of increasingly scientific approaches to competitive training (Chen, 2023), the quantitative assessment and integrated analysis of factors affecting athletic performance have become important components of training-load monitoring and competitive performance optimization (Bourdon et al., 2017). Elite athletic performance is jointly determined by multiple factors, including training stimuli, physiological responses, recovery status, and sport-specific technical proficiency (Mujika et al., 2018; Zentgraf et al., 2024). Sustained, systematic, and scientifically planned training loads can promote the transition from short-term stress responses to long-term adaptation, thereby laying the foundation for athletes to attain an optimal competitive state during competition (Clemente et al., 2025; Lu et al., 2024). Accordingly, an important practical value of training-load monitoring lies in the accurate and dynamic assessment of athletes’ recent competitive status and the subsequent optimization of training arrangements, fatigue management, and recovery strategies (Rebelo et al., 2026).

Training-load monitoring has been widely applied in football (Akenhead and Nassis, 2016; Miguel et al., 2021), basketball (Russell et al., 2021; Petway et al., 2020), and endurance sports (Mujika, 2017; Jobson et al., 2009). Previous studies have primarily examined relationships between training stimuli and performance through external load, internal load, physiological and biochemical markers, and recovery status (Coyne et al., 2018; Scantlebury et al., 2020; Sansone et al., 2023; Lechner et al., 2023; Zhang et al., 2025). Because sports differ substantially in movement demands, energy metabolism, and training organization, the relationship between training load and performance is strongly sport specific (Herring et al., 2019; West et al., 2021).

Training-load monitoring systems should therefore be designed systematically and individualized according to the specific demands of the sport.

Performance in speed climbing depends on strength, speed, and technical proficiency (Askari Hosseini and Wolf, 2023), but may also be influenced by the accumulation and variability of recent training loads and by changes in recovery status (Halson, 2014; Bourdon et al., 2017; Kellmann et al., 2018). As a short-duration, high-intensity sport, speed climbing requires explosive effort. Its training commonly focuses on start reactions, rapid force production, successive pushing and pulling actions, movement-rhythm control, and the ability to complete repeated efforts. These demands expose athletes to substantial physical loading while requiring repeated technical practice (Fuss et al., 2020; Askari Hosseini and Wolf, 2023). Previous studies have mainly examined sport-specific technique, physiological mechanisms, and competition performance. Relatively little research has addressed the relationship between training-load monitoring indicators and competitive performance during the training process (Askari Hosseini and Wolf, 2023).

This study examined six monitoring indicators in elite speed climbers: training strain, training monotony, testosterone, creatine kinase (CK), perceived sleep quality, and soreness. Rolling means and coefficients of variation were used to characterize the recent levels and temporal variability of these indicators. Generalized estimating equations (GEE) were used to assess the overall associations between these time-series features and relative composite performance, while quantile regression was further applied to explore whether these associations differed across performance quantiles. The findings may provide useful information for training-load monitoring and training regulation in elite speed climbing.

## 2 Methods

### 2.1 Study design and participants

Nine elite speed climbers were included in this longitudinal observational study. According to the participant classification framework proposed by McKay et al. (2022), all athletes were classified as Tier 4 or above, corresponding to elite/international-level athletes or higher. The study involved a 30-week monitoring period, which was organized into 15 consecutive two-week monitoring periods. Training-load and subjective recovery measures were collected throughout the entire monitoring period, whereas testosterone and creatine kinase (CK) were generally measured at approximately two-week intervals. All data were derived from the team’s established routine athlete-monitoring system and were subsequently compiled into an analytical dataset for time-series feature extraction and further analysis.

The study was approved by the China Institute of Sport Science (project code: 20260212). All participants agreed to the use of their training-monitoring data for research purposes. The study was conducted in accordance with the Declaration of Helsinki and its subsequent amendments.

### 2.2 Relative composite performance

To examine the association between time-series characteristics of training monitoring and between-athlete differences in relative composite competitive performance, athletes were evaluated using a relative composite performance score (perf score). This score was derived from an integrated assessment of each athlete’s competitive performance within their respective group during the study period, with world ranking serving as the primary reference, supplemented by rankings from simulated competitions conducted during national-team training camps and coaches’ subjective evaluations. No fixed percentage weights were assigned to the individual components, and the final within-group ranking was used to summarize each athlete’s relative composite competitive performance over the study period.

Male and female athletes were evaluated separately, with four athletes in the female group and five in the male group. Based on the above evaluation procedure, each athlete received a single perf score representing their relative composite competitive performance during the study period. The perf score was not recalculated at each monitoring period; the same athlete-level score was used for all rolling observations contributed by a given athlete. Because the two sex-specific groups differed in size, the final within-group rankings were transformed to a common 0–1 scale using the following equation:


$$perfscore=1-\frac{rank-1}{groupn-1}$$

where *n* represents the number of athletes in the corresponding sex-specific group and *rank* represents the athlete’s final within-group ranking, with the highest-ranked athlete assigned *rank=1*.

### 2.3 Core indicators and measurement procedures

The core indicators were predefined based on theoretical considerations related to training monitoring and recovery assessment rather than being selected solely through statistical screening. Training strain and training monotony were used to characterize training-load features; testosterone and creatine kinase (CK) were included as routinely monitored biochemical indicators; and perceived sleep quality and soreness were used to represent subjective sleep and recovery-related information.

Approximately 30 min after each training session, session rating of perceived exertion (session RPE) was recorded using the CR-10 scale (Borg, 1982). Training load for each session was calculated as session RPE multiplied by training duration in minutes. Training monotony was calculated as the mean daily training load divided by the standard deviation of daily training load, while training strain was calculated as total training load multiplied by training monotony.

Testosterone and CK were generally assessed at approximately two-week intervals using fasting venous blood samples collected in the morning. Whenever feasible, blood sampling was preferentially scheduled after a rest day to minimize the immediate effects of preceding exercise; however, the exact sampling dates could be adjusted according to competition schedules, training arrangements, and team logistics. As part of routine athlete monitoring, biochemical testing was conducted at tertiary Grade A hospitals in China. Testosterone was measured using a chemiluminescence method and expressed in ng/dL, whereas CK activity was determined using a rate method and expressed in U/L.

Subjective recovery monitoring was conducted every morning, including on rest days, through an electronic questionnaire distributed by designated staff. Athletes were instructed to complete the questionnaire as their first task after waking. The questionnaire included sleep duration, perceived sleep quality, soreness, and recovery index. Considering the feasibility of long-term continuous monitoring in routine training and competition settings, as well as the monitoring burden on athletes, daily morning subjective assessments were used to monitor sleep quality. No objective sleep measures, such as polysomnography, were used during the study; therefore, the sleep-related indicator primarily reflected athletes’ perceived sleep quality. Perceived sleep quality was rated on an integer scale from 0 to 10, with higher scores indicating better perceived sleep quality, whereas soreness was rated from 0 to 10, with higher scores indicating greater soreness.

### 2.4 Extraction of time-series features

#### 2.4.1 Missing data handling

In the monitoring dataset, some values were missing for testosterone and CK, with 20 missing observations for testosterone (14.8%) and 20 for CK (14.8%). No missing values were present at the monitoring-period level for training strain, training monotony, perceived sleep quality, or soreness.

Missing values were imputed using chained equations combined with predictive mean matching (PMM) (White et al., 2011; van Buuren, 2018), implemented in Python with MICE Data. The imputation dataset included athlete ID, monitoring period, perf score, and the six core monitoring indicators. The PMM donor pool was set to the 20 nearest neighbors (k pmm = 20). After 20 complete chained-equation updating cycles, the final completed dataset was retained for subsequent analyses. Accordingly, Rubin’s rules for pooling across multiple imputed datasets were not applied. All time-series features were calculated after completion of the missing-data procedure.

#### 2.4.2 Time-series feature extraction

Training-load, biochemical, and subjective recovery indicators may change over the course of training, and a single observation may not adequately reflect recent cumulative exposure or the degree of temporal variability (Halson, 2014; Kellmann et al., 2018). Therefore, rolling level and variability features were used to characterize the longitudinal monitoring data.

Time-series features were calculated using a rolling window comprising four consecutive monitoring periods (8 weeks), representing a mesocycle-scale training interval (Issurin, 2008; Stellingwerff et al., 2019). The rolling mean was used to represent the average level of each indicator over the corresponding time interval, whereas the rolling coefficient of variation (CV) was used to characterize variability over the same interval. For the six core monitoring indicators, a total of 12 candidate time-series features were generated.

The rolling window advanced by one monitoring period at each step; consequently, adjacent windows shared three monitoring periods. Because a complete four-period rolling window could not be calculated for the first three monitoring periods, each athlete contributed 12 rolling observations. Thus, the nine athletes provided a total of 108 observations for the time-series analyses.

Rolling Mean

This feature represents the average level of an indicator across four consecutive monitoring periods and was calculated as follows:


$$Rollingmean_{4}=\frac{1}{4}\sum_{k=t-3}^{t}xk$$

Rolling Coefficient of Variation

This feature represents the degree of variability in an indicator across four consecutive monitoring periods and was calculated as follows:


$$RollingCV_{4}=\frac{SD\left(x_{t-3},x_{t-2},x_{t-1},x_{t}\right)}{Mean\left(x_{t-3},x_{t-2},x_{t-1},x_{t}\right)}$$

### 2.5 Statistical analysis

#### 2.5.1 Generalized estimating equation analysis

Generalized estimating equations (GEE) were used to examine the population-averaged associations between the 12candidate time-series features and the relative composite performance score (perf score). A separate GEE model was fitted for each candidate feature, with athlete ID specified as the clustering variable. The primary analysis used a Gaussian family with an identity link and an independence working correlation structure, together with athlete-clustered robust covariance estimation. Regression coefficients (β), 95% confidence intervals (95% CIs), P values, and false discovery rate (FDR)-adjusted P values were reported. GEE served as the primary approach for estimating population-averaged associations, whereas quantile regression was used as a complementary analysis to explore whether these associations differed across performance quantiles.

#### 2.5.2 Univariable quantile regression analysis

Exploratory univariable quantile regression analyses were performed separately for each of the 12candidate time-series features. Regression coefficients were estimated across τ = 0.10–0.90, with particular emphasis on τ = 0.25, 0.50, and 0.75, representing the lower, median, and upper positions of the conditional distribution of the relative composite performance score, respectively. Because each athlete contributed multiple repeated observations, robust covariance estimation was performed using athlete ID as the clustering unit. The univariable analyses were primarily used to compare the direction and magnitude of associations for each candidate time-series feature across different quantiles and to inform the subsequent multivariable quantile regression analyses.

#### 2.5.3 Multivariable quantile regression: main and extended models

Following the univariable analyses, an exploratory multivariable quantile regression model was developed. For the main model, one time-series feature was chosen to represent each of the six core monitoring indicators, resulting in six predictors. Selection of these variables was guided by their theoretical relevance, the findings from the univariable analyses and GEE, the results of collinearity assessment, and the need to maintain a parsimonious model. The final set of predictors comprised the rolling mean of training strain, the rolling mean of training monotony, the rolling mean of testosterone, the rolling mean of CK, the rolling CV of perceived sleep quality, and the rolling mean of soreness.

Both models were estimated at τ = 0.25, 0.50, and 0.75, with interpretation based primarily on the six-predictor main model. The extended model contained the full set of 12 time-series features and served as an exploratory sensitivity analysis to assess whether the main associations were sensitive to model specification. For descriptive visualization, the six-predictor main model was additionally estimated across τ = 0.10–0.90 in increments of 0.05.

#### 2.5.4 Collinearity diagnostics and multiple-comparison correction

Variance inflation factors (VIFs) were used to assess collinearity among predictors in the multivariable models. For the GEE analyses, false discovery rate (FDR) correction was applied to the P values from the 12candidate time-series features. For the quantile regression analyses, FDR correction was performed separately within each quantile, with 12, 6, and 12 P values adjusted for the univariable models, six-predictor main model, and 12-predictor extended model, respectively. For both GEE and quantile regression, regression coefficients (β), 95% confidence intervals (95% CIs), unadjusted P values, and FDR-adjusted P values were reported. All statistical tests were two-sided, and all analyses were conducted in Python.

#### 2.5.5 Robustness and sensitivity analyses

Because of the small number of athletes, additional robustness and sensitivity analyses were conducted. Convergence of the GEE and quantile regression models was examined. For GEE, exchangeable and AR(1) working correlation structures were additionally fitted to assess the sensitivity of the results to the specification of the within-athlete correlation structure. Small-cluster bias-reduced covariance estimation was also applied to evaluate the robustness of statistical inference under the limited number of athlete clusters.

In addition, leave-one-athlete-out (LOAO) analyses were performed by sequentially excluding one athlete and refitting the GEE models and the six-predictor main quantile regression model at τ = 0.25, 0.50, and 0.75. Predictors showing marked right-skewness were log10-transformed, after which the main models were refitted to assess the sensitivity of the findings to the influence of individual athletes and to variable scaling.

## 3 Results

### 3.1 Participants and data characteristics

**Figure 1:**
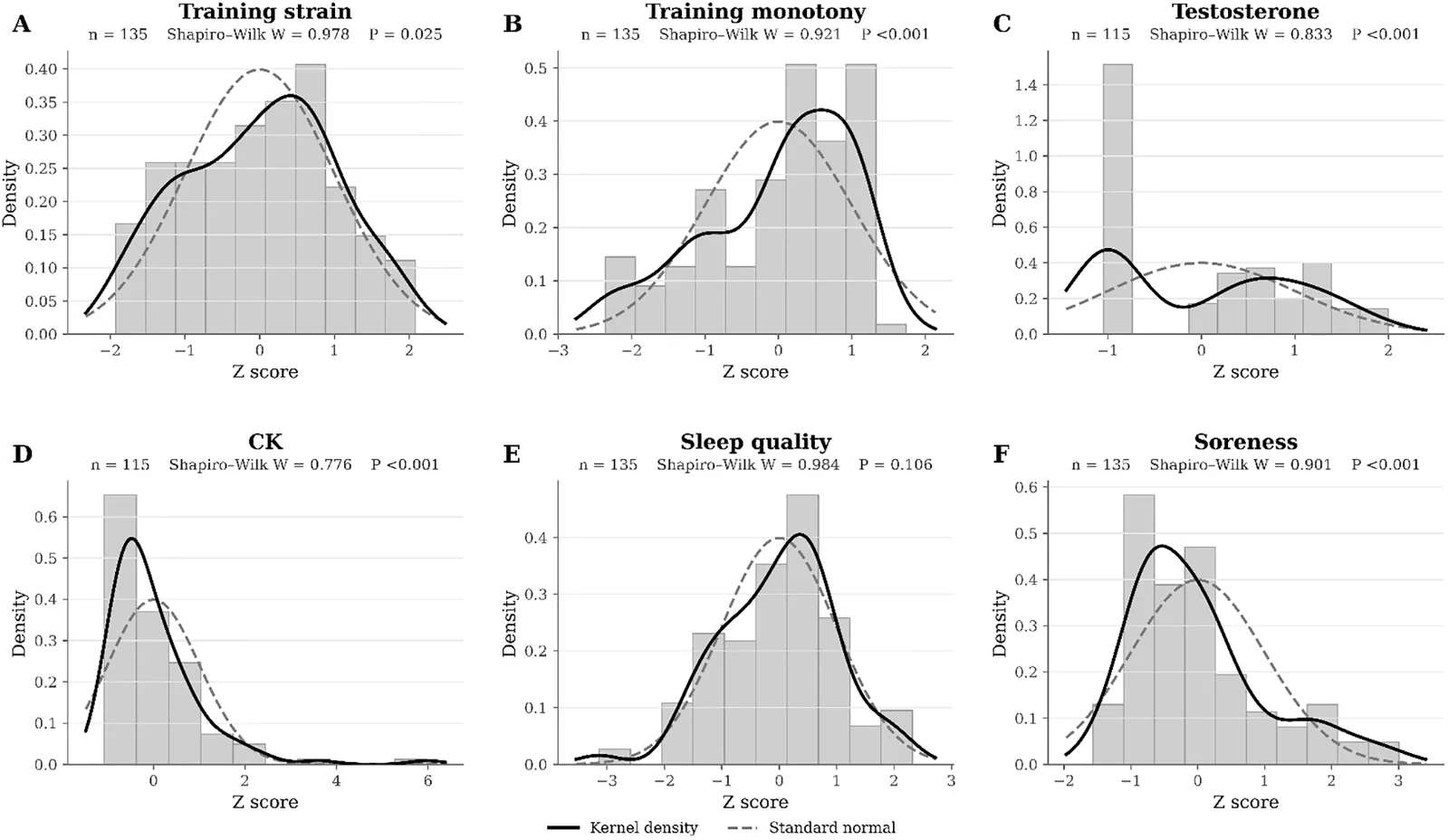
Distributions of the six core training-monitoring indicators: **(A)** training strain, **(B)** training monotony, **(C)** testosterone, **(D)** creatine kinase (CK), **(E)** perceived sleep quality, and **(F)** soreness. Histograms show the pre-imputation observations with kernel density curves. Dashed curves indicate the standard normal distribution. Shapiro–Wilk results are descriptive only.

A total of nine athletes were included in the study, providing repeated monitoring data across 15 consecutive monitoring periods. The analysis included six core monitoring indicators: training strain, training monotony, testosterone, creatine kinase (CK), perceived sleep quality, and soreness. The distributions of the raw observations for these indicators are presented in **Figure 1**. All indicators showed some degree of variability over the monitoring period, with testosterone and CK exhibiting relatively greater dispersion. The Shapiro–Wilk test indicated that perceived sleep quality was approximately normally distributed, whereas training strain, training monotony, testosterone, CK, and soreness showed non-normal distributions.

### 3.2 GEE results

**Figure 2:**
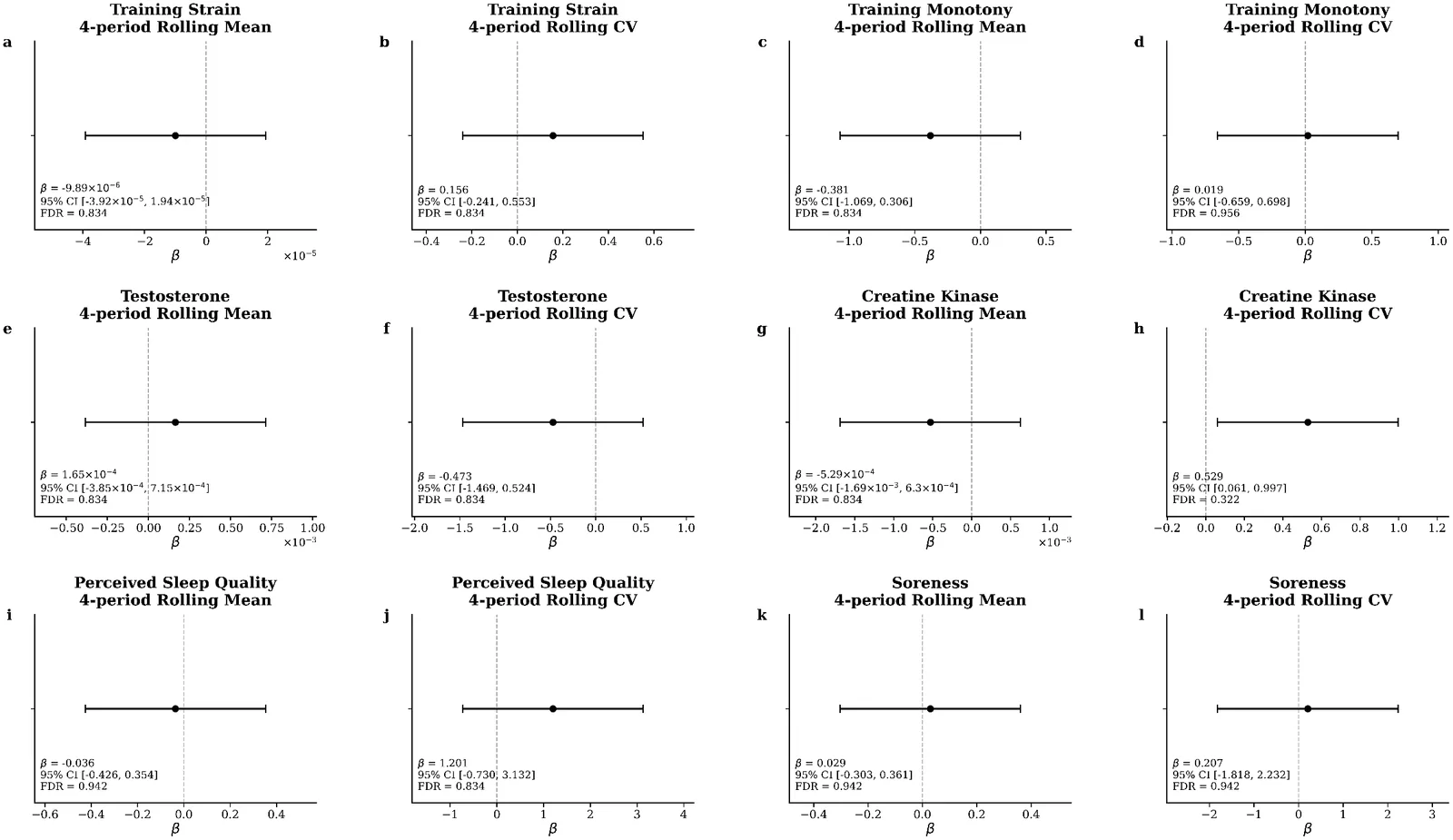
Forest plots of the primary GEE estimates for the 12 candidate time-series features: **(a)** training strain rolling mean, **(b)** training strain rolling CV, **(c)** training monotony rolling mean, **(d)** training monotony rolling CV, **(e)** testosterone rolling mean, **(f)** testosterone rolling CV, **(g)** CK rolling mean, **(h)** CK rolling CV, **(i)** perceived sleep quality rolling mean, **(j)** perceived sleep quality rolling CV, **(k)** soreness rolling mean, and **(l)** soreness rolling CV. Points indicate GEE coefficients (β), and horizontal bars indicate athlete-clustered robust 95% CIs. The dashed line indicates β = 0. FDR correction was applied across the 12 tests.

Among the 12candidate time-series features, only CK rolling CV showed a nominal positive association with the relative composite performance score (β = 0.529, 95% CI 0.061–0.997, P = 0.027). However, this association was no longer statistically significant after FDR correction (FDR = 0.322). None of the remaining 11 time-series features reached statistical significance based on either the unadjusted P values or the FDR-adjusted results. Overall, the primary GEE analysis did not identify any stable population-averaged associations that remained statistically significant after correction for multiple comparisons (**Figure 2**; **Supplementary Table 1**).

### 3.3 Univariable quantile regression results

The univariable quantile regression analyses showed that the direction and magnitude of the regression coefficients varied across the conditional quantiles of the relative composite performance score. Complete results for τ = 0.25, 0.50, and 0.75 are provided in **Supplementary Table 2**, and the numerical results across the continuous quantile range are presented in **Supplementary Table 3**.

At τ = 0.25, testosterone rolling mean was positively associated with perf score (β = 5.259 × 10^-4^, FDR = 0.002), whereas CK rolling mean showed a negative association (β = −8.315 × 10^-4^, FDR = 0.005). CK rolling CV and perceived sleep-quality rolling CV were both positively associated with perf score (β = 0.535 and 1.597, respectively; both FDR < 0.01). None of the remaining features reached statistical significance after FDR correction.

At τ = 0.50, testosterone rolling CV was negatively associated with perf score (β = −0.660, FDR = 0.022), and CK rolling mean also showed a negative association (β = −9.307 × 10^-4^, FDR = 0.004). CK rolling CV, perceived sleep-quality rolling CV, and soreness rolling mean were positively associated with perf score, with β values of 0.561, 2.199, and 0.245, respectively (all FDR < 0.01).

At τ = 0.75, training strain rolling mean was negatively associated with perf score (β = −2.715 × 10^-5^, FDR = 0.002), whereas its rolling CV was positively associated (β = 0.366, FDR < 0.001). Training monotony rolling mean showed a negative association (β = −0.868, FDR < 0.001), while its rolling CV showed a positive association (β = 0.934, FDR < 0.001). Testosterone rolling CV was negatively associated with perf score (β = −1.453, FDR = 0.001). In addition, CK rolling CV, perceived sleep-quality rolling CV, soreness rolling mean, and soreness rolling CV were positively associated with perf score, with β values of 0.665, 2.135, 0.169, and 1.737, respectively (all FDR < 0.05).

CK rolling CV and perceived sleep-quality rolling CV were positively associated with perf score across all three representative quantiles. Significant associations for the other indicators were mainly observed at specific quantiles, suggesting that the direction or magnitude of these associations may vary across the conditional distribution of relative composite performance.

### 3.4 Multivariable quantile regression and collinearity diagnostics

In the main model, training monotony rolling mean, testosterone rolling mean, CK rolling mean, and perceived sleep-quality rolling CV showed consistent directions of association at τ = 0.25, 0.50, and 0.75 and remained statistically significant after FDR correction. Specifically, training monotony rolling mean was consistently negatively associated with perf score, with β values of −1.351, −1.516, and −0.886, respectively. Testosterone rolling mean showed consistent positive associations (β = 4.386 × 10^-4^, 4.427 × 10^-4^, and 6.039 × 10^-4^), whereas CK rolling mean showed consistent negative associations (β = −7.243 × 10^-4^, −7.823 × 10^-4^, and approximately −0.001). Perceived sleep-quality rolling CV was consistently positively associated with perf score (β = 1.748, 1.972, and 1.587, respectively; all FDR < 0.01).

**Figure 3:**
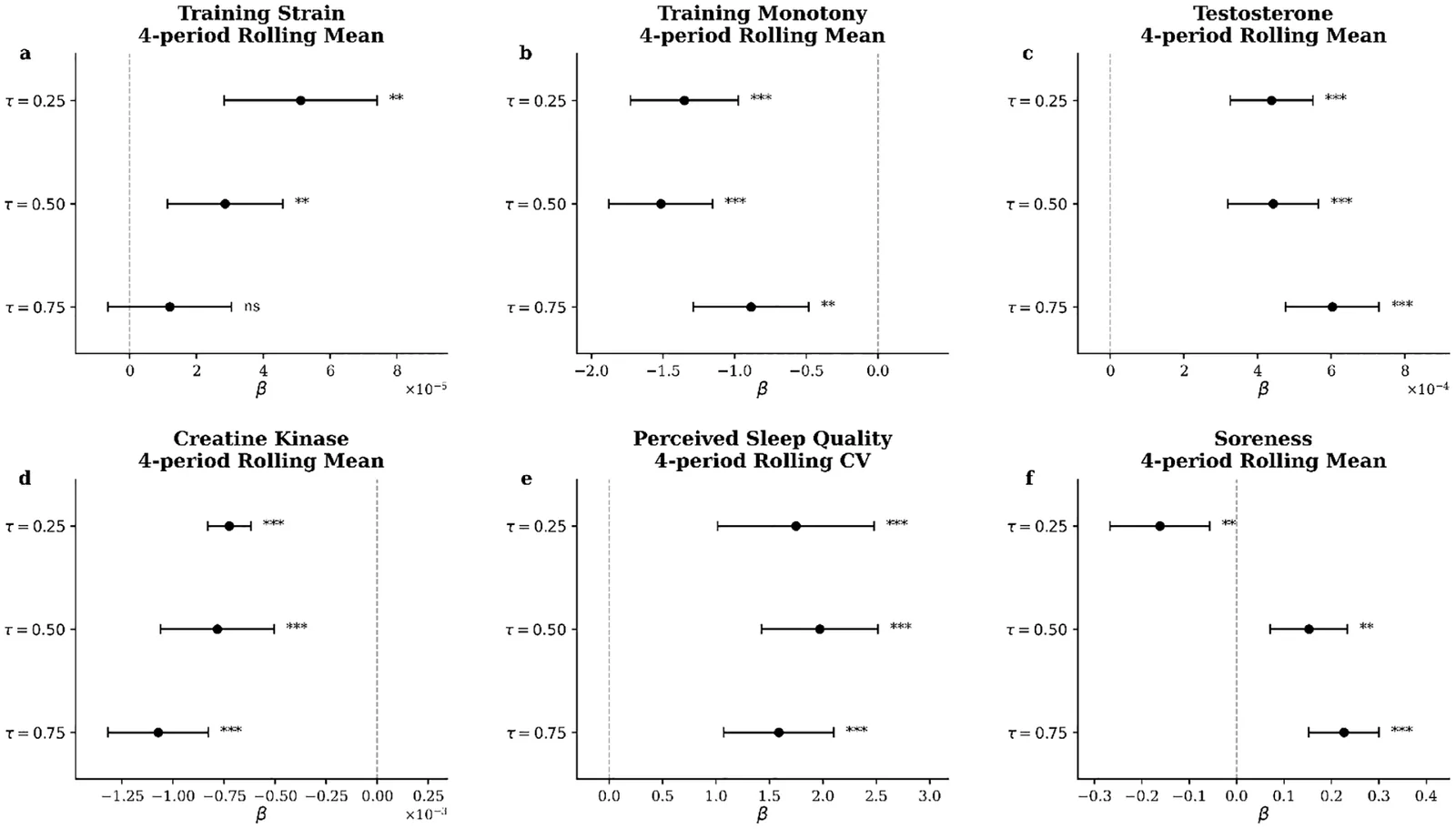
Multivariable quantile regression estimates for the six-predictor main model: **(a)** training strain rolling mean, **(b)** training monotony rolling mean, **(c)** testosterone rolling mean, **(d)** CK rolling mean, **(e)** perceived sleep quality rolling CV, and **(f)** soreness rolling mean, at τ = 0.25, 0.50, and 0.75. Black circles indicate coefficient estimates (β), and error bars indicate athlete-clustered robust 95% CIs at τ = 0.25, 0.50, and 0.75. Significance is based on FDR-adjusted P values (**FDR < 0.01, *FDR < 0.001; ns, FDR ≥ 0.05).

**Figure 4:**
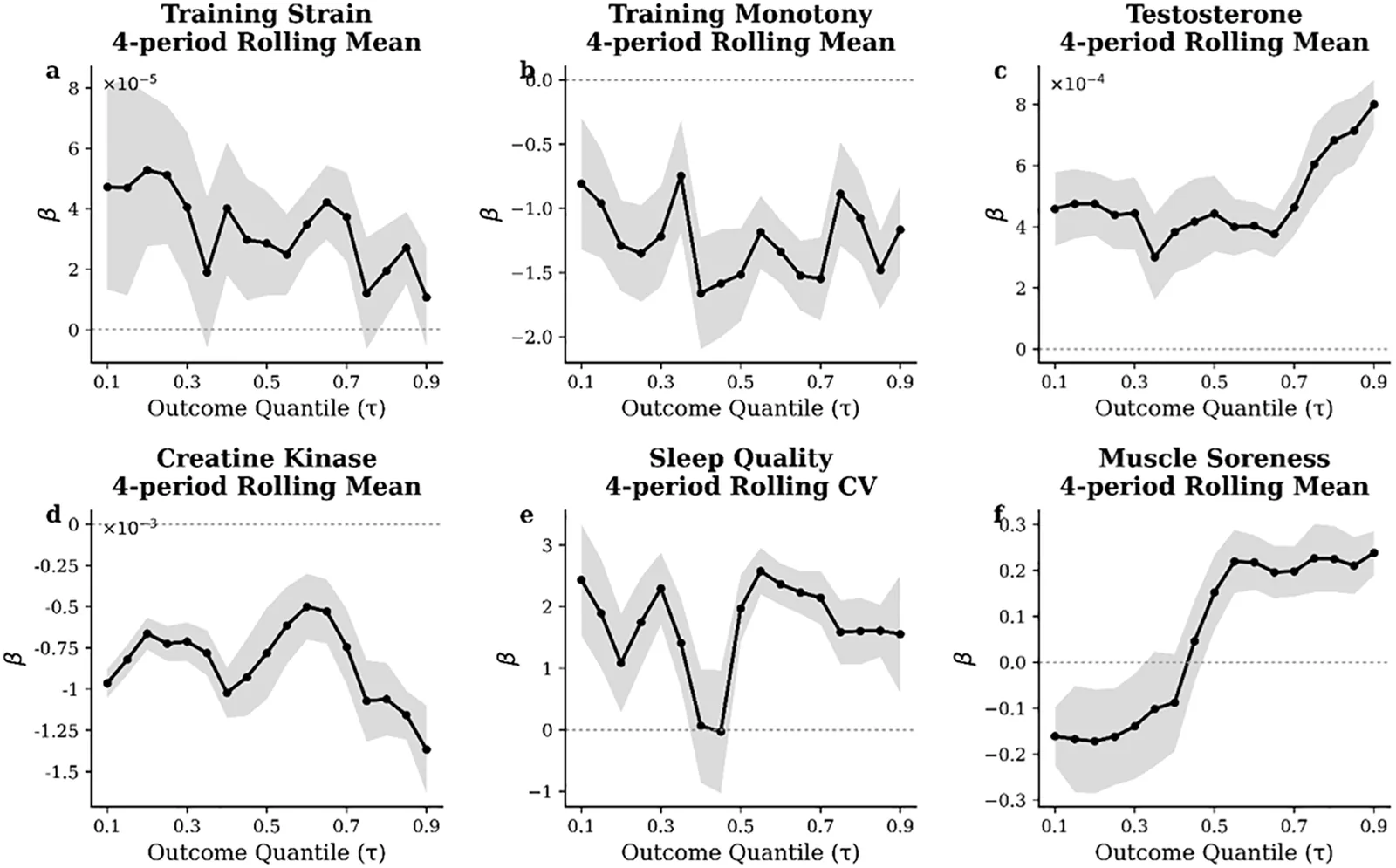
Quantile-specific coefficient profiles across the conditional distribution of relative composite performance for **(a)** training strain rolling mean, **(b)** training monotony rolling mean, **(c)** testosterone rolling mean, **(d)** CK rolling mean, **(e)** perceived sleep quality rolling CV, and **(f)** soreness rolling mean. Solid lines show quantile-regression coefficients across τ = 0.10–0.90, with shaded 95% CIs. The dashed line indicates β = 0.

Training strain rolling mean was positively associated with perf score at τ = 0.25 and 0.50 (β = 5.121 × 10^-5^ and 2.861 × 10^-5^, respectively; both FDR < 0.01), but the association was not statistically significant at τ = 0.75 (β = 1.203 × 10^-5^, FDR = 0.171). The direction of the association for soreness rolling mean varied across quantiles. It was negatively associated with perf score at τ = 0.25 (β = −0.162, FDR = 0.007) but became positively associated at τ = 0.50 and 0.75 (β = 0.152 and 0.227, respectively; both FDR < 0.01). (**Figures 3**, **4**; **Supplementary Table 4**).

In the extended model, the negative association for training monotony rolling mean, the positive association for testosterone rolling mean, and the negative association for CK rolling mean remained directionally consistent across all three representative quantiles. Perceived sleep-quality rolling CV also remained positively associated with perf score. In contrast, the directions of some additional rolling CV features varied across quantiles, indicating a degree of sensitivity to model specification. The extended model was therefore interpreted primarily as an exploratory sensitivity analysis. (**Supplementary Table 5**).

**Figure 5:**
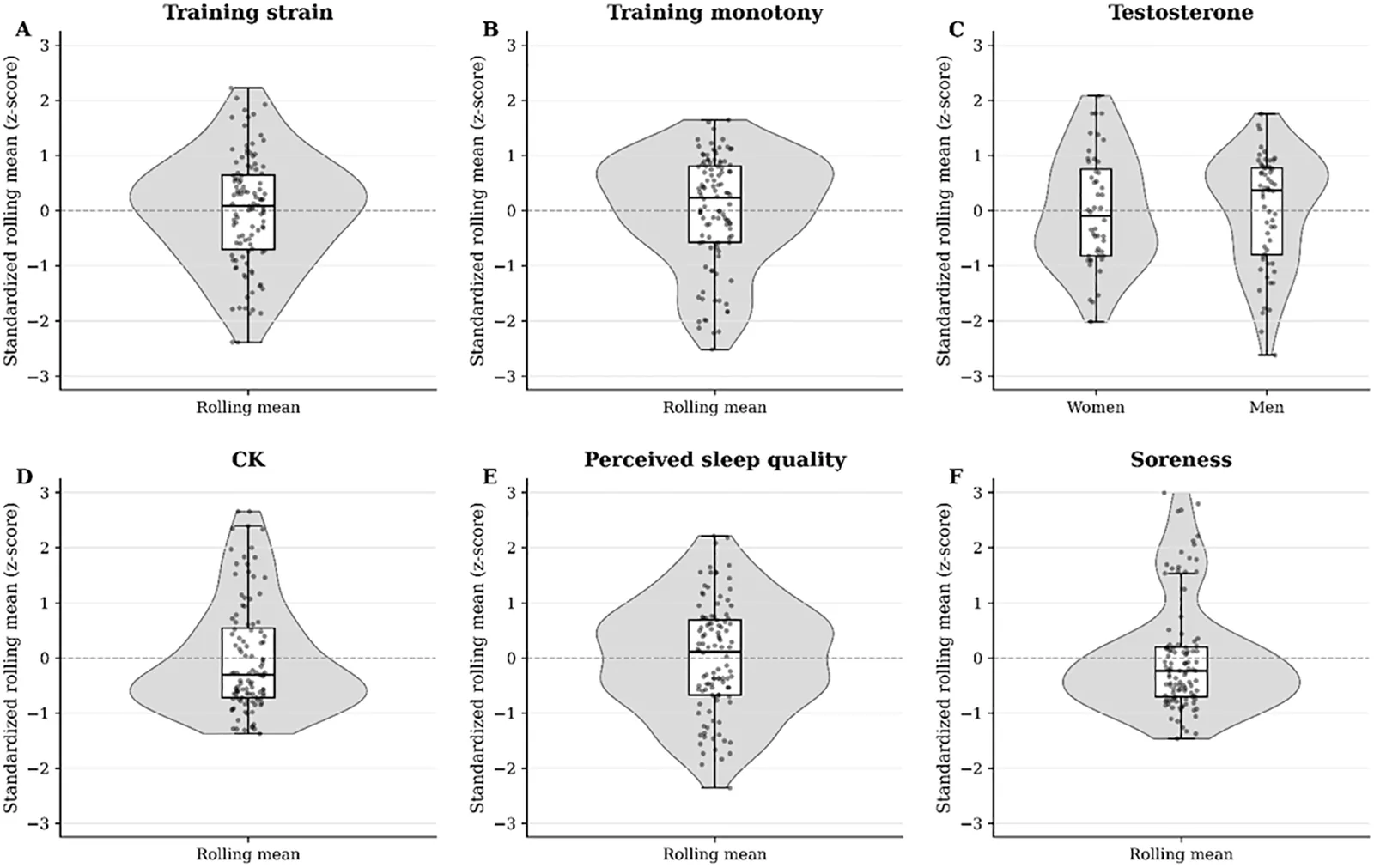
Distributions of standardized rolling means for the six core monitoring indicators: **(A)** training strain, **(B)** training monotony, **(C)** testosterone, **(D)** CK, **(E)** perceived sleep quality, and **(F)** soreness. Violin plots show the distributions of standardized rolling means; boxplots indicate the median and interquartile range, and points represent pooled rolling observations. Testosterone is displayed separately for women and men.

Collinearity diagnostics showed that VIFs in the main model ranged from 1.084 to 7.314. The VIFs for training strain rolling mean and training monotony rolling mean were 7.314 and 6.709, respectively, whereas those for the remaining predictors were below 2. In the extended model, VIFs ranged from 1.159 to 9.536. The VIFs for training monotony rolling mean, training strain rolling mean, training monotony rolling CV, and training strain rolling CV were 9.536, 9.425, 8.215, and 7.724, respectively, while those for all other features were below 3. These findings indicated relatively substantial information overlap among the time-series features related to training strain and training monotony (**Figure 5**; **Supplementary Tables 6**, **7**).

### 3.5 Robustness and sensitivity analyses

The statistical evidence for the population-averaged associations estimated by GEE was sensitive to small-cluster adjustment. Both the primary independence GEE and the exchangeable working correlation structure converged successfully, with the latter yielding an estimated within-athlete correlation coefficient close to 1 (ρ ≈ 0.985), whereas the AR(1) structure did not produce stable estimates. After applying small-cluster bias-reduced covariance estimation, the regression coefficient for CK rolling CV remained positive (β = 0.529), but the 95% CI widened from 0.061–0.997 under the conventional robust estimate to −0.814–1.872, with P = 0.440, indicating that the nominal statistical significance was not retained after small-cluster adjustment.

The leave-one-athlete-out analyses further showed that, in the GEE models, both CK rolling mean and CK rolling CV retained their original directions in all 9 analyses. In the six-predictor main quantile regression model, CK rolling mean retained the same direction in 9/9 analyses at τ = 0.25, 0.50, and 0.75. The corresponding proportions were 8/9, 9/9, and 9/9 for training monotony rolling mean, and 9/9, 8/9, and 9/9 for testosterone rolling mean. In comparison, the directional stability of perceived sleep-quality rolling CV and soreness rolling mean was relatively lower.

After log10 transformation of markedly right-skewed predictors, the negative association for training monotony rolling mean, the positive association for testosterone rolling mean, and the negative association for CK rolling mean remained directionally consistent across all three representative quantiles in the main model. Perceived sleep-quality rolling CV changed direction at τ = 0.50. In the GEE analysis, CK rolling CV remained positively associated on the log10 scale (β = 0.539, FDR = 0.025), although the FDR-adjusted P value increased to 0.319 after small-cluster bias correction.

Convergence diagnostics showed that the six-predictor main model converged successfully at all three prespecified quantiles of τ = 0.25, 0.50, and 0.75. Some convergence limitations were observed at selected quantiles in the continuous-quantile analyses and in the 12-feature extended model. Complete results for alternative working correlation structures, small-cluster adjustment, LOAO analyses, variable-scale transformation, and model convergence diagnostics are provided in the **Supplementary Material**. (**Supplementary Tables 8**–**14**).

## 4 Discussion

This study combined GEE and quantile regression to explore the associations between time-series features of training-load, biochemical, and subjective recovery indicators and relative composite performance in elite speed climbers from two complementary perspectives: population-averaged associations and different positions of the conditional distribution of the relative composite performance score. (1)The GEE analysis did not identify stable population-averaged associations with consistent statistical support, suggesting limited evidence for a uniform linear association at the population-average level. (2) Exploratory quantile regression showed that training monotony rolling mean was generally negatively associated with relative composite performance across the representative conditional quantiles, whereas testosterone rolling mean was generally positively associated and CK rolling mean was generally negatively associated. These directional patterns showed relatively high consistency across the main model, extended model, leave-one-athlete-out analyses, and log10 scale sensitivity analyses. In contrast, the findings for perceived sleep-quality rolling CV and soreness were less stable, with their direction or magnitude varying across model specifications, exclusion of individual athletes, or changes in variable scale. (3) These findings suggest that time-series characteristics of training-load, biochemical, and recovery indicators may show heterogeneous associations with between-athlete differences in relative composite performance. Such heterogeneity may not be fully captured by a single population-averaged effect.

### 4.1 Population-averaged and conditional quantile associations

GEE and quantile regression capture different dimensions of the association structure. GEE is used to estimate population-averaged associations, whereas quantile regression evaluates associations at specific quantiles of the conditional outcome distribution, allowing assessment of whether the relationship between predictors and the outcome differs across positions of that distribution (Koenker, 2017). In this study, no stable population-averaged associations remained after FDR correction in the GEE analyses. By comparison, quantile regression indicated that the direction and magnitude of associations for several training-monitoring indicators varied across the conditional distribution of the relative composite performance score. Some indicators showed relatively consistent directional patterns across multiple representative quantiles, whereas others exhibited associations that were specific to particular quantiles. These results suggest that the relationship between training-monitoring indicators and relative composite performance may not be uniform across the conditional outcome distribution. Accordingly, a single population-averaged estimate may not capture the full pattern of associations, and quantile regression offers complementary information to the population-averaged analysis.

From the practical perspective of training monitoring, such heterogeneous association patterns are plausible. Elite athletes may differ substantially in training exposure, recovery capacity, and physiological responses, meaning that the same training stimulus or monitoring indicator may not have identical implications across athletes (Halson, 2014; Bourdon et al., 2017; Kellmann et al., 2018). Therefore, examining associations at different positions of the conditional outcome distribution, in addition to population-averaged associations, may provide complementary information on the heterogeneous association patterns that may exist in training-monitoring data from elite athletes.

### 4.2 Training monotony, training strain, and training-load organization

Training monotony was one of the training-load characteristics that showed a relatively consistent direction of association in this study. Its rolling mean was negatively associated with relative composite performance at all three representative conditional quantiles and showed good directional consistency across the main model, extended model, and sensitivity analyses. In contrast, the direction of the associations for training strain varied more noticeably across quantiles and model specifications. These findings suggest that, in addition to the overall amount of training load, the way training load is organized and varied over a given period may also represent an important training characteristic associated with relative composite performance.

Training monotony and training strain are both derived from session-RPE–based training-load measures, but they emphasize different aspects of the training process. Training monotony primarily reflects the degree of variation in training load, with higher values indicating smaller differences in load across consecutive training days. Training strain is determined jointly by total training load and training monotony and therefore incorporates information on both training volume and load organization. When introducing the concepts of training monotony and training strain, Foster (1998) proposed that training stress is related not only to the total amount of training load but also to its temporal distribution and variation. Subsequent research on training-load monitoring has further emphasized that the training stimulus experienced by athletes should be understood from multiple perspectives, including training volume, intensity, and temporal organization (Foster et al., 2017; Bourdon et al., 2017). Because training monotony is directly incorporated into the calculation of training strain, the two measures share part of the same underlying information. The relatively high VIF values observed in this study also reflect this structural relationship. Strong information overlap among predictors can increase uncertainty in regression coefficient estimates and reduce the ability of a model to distinguish the independent association of each variable (Dormann et al., 2013). Therefore, the greater variation in the direction of training-strain associations across conditional quantiles and model specifications may reflect both its combined representation of total training load and training monotony and the information overlap between these derived measures. The relatively stable negative association observed for training monotony further highlights the potential informational value of the temporal organization of training load in long-term athlete monitoring.

This finding is also relevant in the specific context of speed-climbing training. Speed-climbing programs typically combine strength and power development, sport-specific technical practice, and repeated route training (Askari Hosseini and Wolf, 2023; Muñoz de la Cruz et al., 2024; Pandurević et al., 2025). Repeated practice on the standardized route can help athletes establish stable movement patterns and improve sport-specific technical proficiency. However, from the perspective of training-load organization, prolonged exposure to highly repetitive training content may also make the training stimulus increasingly similar over time. Therefore, while repeated sport-specific practice remains necessary in speed climbing, appropriate variation in training load, training content, and forms of training stimulus may also warrant attention when organizing training.

### 4.3 Association patterns of biochemical and subjective indicators

Testosterone and CK were the two biochemical indicators that showed relatively consistent directions of association in this study, although they reflect different types of physiological information. Testosterone rolling mean was generally positively associated with relative composite performance across the three representative conditional quantiles, whereas CK rolling mean was generally negatively associated. These directional patterns remained relatively consistent in both the main model and the principal sensitivity analyses. Testosterone and CK reflect different physiological aspects of the training process. Testosterone is involved in protein metabolism, muscle-tissue remodeling, and exercise-related endocrine responses, and is influenced by multiple factors, including training volume and intensity, energy status, recovery status, and sampling conditions. CK is widely used as a biochemical marker of skeletal-muscle responses to exercise and can be affected by exercise mode, characteristics of muscle contraction, recent training load, sampling timing, and individual differences (Crewther et al., 2006; Brancaccio et al., 2007; Bourdon et al., 2017). In particular, elevated CK may reflect maladaptation to training, but it may also represent varying degrees of skeletal-muscle response following recent exercise stimuli (Brancaccio et al., 2007). Therefore, changes in testosterone and CK should be interpreted in conjunction with the specific training and recovery context as well as the conditions under which samples were collected.

Soreness rolling mean showed a negative association at the lower conditional quantile but shifted to positive associations at the median and upper conditional quantiles. Its directional stability in the leave-one-athlete-out analyses was also lower than that observed for training monotony, testosterone, and CK. Soreness is influenced by multiple factors, including recent training exposure, muscular load, recovery conditions, individual tolerance, and psychological perception (Halson, 2014; Saw et al., 2016; Kellmann et al., 2018). Accordingly, its relationship with competitive performance may not follow a simple and uniform linear pattern. Perceived sleep-quality rolling CV also showed some sensitivity to model specification, with the direction of association changing at certain conditional quantiles after scale transformation, indicating relatively limited stability of this pattern. Subjective indicators may provide monitoring information that complements training-load and biochemical measures, but their interpretation is more appropriately made in conjunction with the specific training context and other monitoring indicators.

### 4.4 Complementary information from the mean level and variability of time-series features

Training-monitoring research has increasingly emphasized the importance of continuous longitudinal observation rather than relying on isolated measurements. Athletes’ training-load, physiological, and recovery indicators may vary in response to training stimuli, recovery arrangements, and other contextual factors. Therefore, interpretation of monitoring indicators should consider not only their average levels but also their temporal variation over a given training period (Plews et al., 2013; Bourdon et al., 2017). From this perspective, the rolling mean reflects the relatively sustained average level of an indicator over a defined period, whereas the rolling CV reflects the degree of variability relative to its mean during the same period. These two features characterize different temporal properties of the same monitoring series and may provide a more comprehensive description of changes in training-monitoring indicators over time.

In the present study, the rolling mean and rolling CV of some indicators showed different association patterns. For example, CK rolling mean and CK rolling CV showed associations in different directions, while the variability of perceived sleep quality also demonstrated some sensitivity to variable scaling. These findings further suggest that the average level of a given indicator over a training period may not be sufficient to fully characterize its longitudinal variation. The value of time-series features may therefore lie more in their integrated interpretation alongside multidimensional monitoring information on training load, biochemical responses, and subjective recovery. Future studies incorporating repeated objective performance outcomes should further examine whether rolling mean and variability features provide complementary and reproducible longitudinal information, and whether these features demonstrate stable within-athlete associations and predictive value.

### 4.5 Methodological considerations and study limitations

The analytical framework of this study focuses simultaneously on the overall average association between training monitoring indicators, differences in conditional distributions, and temporal variation characteristics. Generalized Estimating Equations (GEE) were employed to describe the overall average association between candidate time-series features and relative competitive performance, whilst quantile regression was used to further explore whether this association varied across different points in the conditional distribution of the outcome; rolling means and rolling CV summarized the average levels and relative variability of the indicators within an 8-week time window, respectively. Consequently, training load, biochemical and subjective recovery information could be characterized from various perspectives, including average levels, temporal variability and conditional distributions. Given the limited number of athletes, this study further evaluated the stability of the results by employing different GEE working-related structures, small-cluster bias-reduced covariance estimation, LOAO, log10 scaling and model convergence checks; the 6-predictor model was used as the primary exploratory multivariate model, while the 12-feature extended model served as a sensitivity analysis.

The main limitations of this study relate to the data structure and the limited number of independent observations and can be summarized in five aspects. (1) Independent sample size and model complexity. Rolling calculations across 15 monitoring periods generated 108 analytical observations; however, all observations were derived from only nine athletes. Repeated measurements increased the amount of longitudinal monitoring information but did not increase the number of independent individuals, and the precision of statistical inference therefore remained constrained by the limited sample size. Although all participants were Tier 4 or higher elite athletes and thus represented a highly specialized sample, the six-predictor main model, and particularly the 12-feature extended model, remained relatively complex in relation to the nine independent athletes. Some findings may therefore have been sensitive to individual athletes, variable scaling, and model specification. (2) Data level of the outcome variable. Each athlete had only one relative composite performance score (perf score) over the study period. Accordingly, the current models primarily reflect associations between long-term monitoring characteristics and between-athlete differences in relative composite performance and cannot determine whether changes in an individual athlete’s monitoring indicators relative to their own baseline correspond to concurrent changes in competitive performance. (3) Overlap of rolling windows. Adjacent rolling windows shared three monitoring periods, resulting in substantial information overlap and temporal dependence between consecutive observations. The current analyses did not explicitly account for all serial correlation arising from this overlap. In addition, different rolling-window lengths were not compared; therefore, the sensitivity of the findings to the selected four-monitoring-period window cannot be determined. Future studies should evaluate the findings using alternative window lengths. (4) Missing-data handling and subjective recovery measurement. Missing testosterone and CK values were handled using chained equations combined with PMM; however, only one completed dataset was retained, and Rubin’s rules were not used to pool estimates across multiple imputed datasets. Thus, imputation uncertainty was not fully incorporated into the final statistical inference. Perceived sleep quality was derived from morning self-reports and therefore reflects athletes’ subjective perceptions of sleep, which are not fully equivalent to the information provided by objective sleep-monitoring methods (Sterne et al., 2009). (5) Study design. Because this was an observational study, the identified relationships represent statistical associations between training-monitoring features and relative composite performance and cannot be interpreted as causal effects.

## 5 Conclusion

This study examined the associations between time-series features of training-load, biochemical, and subjective recovery indicators and relative composite performance using longitudinal monitoring data from elite speed climbers. After correction for multiple comparisons and small-cluster sensitivity analyses, GEE did not identify stable population-averaged associations. In contrast, quantile regression indicated that different monitoring indicators showed different association patterns across the conditional distribution of relative composite performance. Training monotony rolling mean was generally negatively associated with relative composite performance, testosterone rolling mean was generally positively associated, and CK rolling mean was generally negatively associated. These directional patterns showed relatively good consistency across the main model and sensitivity analyses. In comparison, features related to training strain, soreness, and perceived sleep quality showed greater variation across model specifications or conditional quantiles.

The different association patterns observed for rolling means and rolling CVs suggest that the average level and temporal variability of monitoring indicators may provide complementary longitudinal information. Given the limited number of independent athletes, the athlete-level nature of the perf score, which primarily reflects between-athlete differences in relative composite performance, and the observational study design, these findings should be regarded as exploratory and hypothesis-generating. Future studies should include larger samples of elite athletes, incorporate repeatedly measured objective sport-specific performance outcomes, and apply longitudinal analytical approaches capable of distinguishing within-athlete from between-athlete effects. Such work is needed to determine the stability of these time-series features and their potential value for training monitoring and performance prediction.

## Data Availability

The original contributions presented in the study are included in the article/**Supplementary Material**. Further inquiries can be directed to the corresponding authors.
