# Supplementary material for: Associations between time-series features of training-load monitoring indicators and relative composite performance in elite speed climbers: a GEE and quantile regression study
Source: Front Physiol. 2026 Sep 11;17:1959824. doi: 10.3389/fphys.2026.1959824 (PMC13613050; doi:10.3389/fphys.2026.1959824)
Supplement: Supplementary file 1 [file DataSheet1.pdf]

Supplementary Tables

Supplementary Table S1. Primary athlete-clustered robust GEE results for the 12 candidate time-series features.

Feature	n	No. of athletes	β	95% CI	P value	FDR-adjusted P value
Training strain 4 period rolling mean (8 weeks)	108	9	-9.889×10^{-6}	-3.920×10^{-5} to 1.943×10^{-5}	0.509	0.834
Training strain 4 period rolling CV (8 weeks)	108	9	0.156	-0.241 to 0.553	0.440	0.834
Training monotony 4 period rolling mean (8 weeks)	108	9	-0.381	-1.069 to 0.306	0.277	0.834
Training monotony 4 period rolling CV (8 weeks)	108	9	0.019	-0.659 to 0.697	0.956	0.956
Testosterone 4 period rolling mean (8 weeks)	108	9	1.651×10^{-4}	-3.846×10^{-4} to 7.149×10^{-4}	0.556	0.834
Testosterone 4 period rolling CV (8 weeks)	108	9	-0.473	-1.469 to 0.524	0.352	0.834
CK 4 period rolling mean (8 weeks)	108	9	-5.291×10^{-4}	-0.002 to 6.299×10^{-4}	0.371	0.834
CK 4 period rolling CV (8 weeks)	108	9	0.529	0.061 to 0.997	0.027	0.322
Perceived sleep quality 4 period rolling mean (8 weeks)	108	9	-0.036	-0.426 to 0.354	0.855	0.942
Perceived sleep quality 4 period rolling CV (8 weeks)	108	9	1.201	-0.730 to 3.132	0.223	0.834
Soreness 4 period rolling mean (8 weeks)	108	9	0.029	-0.303 to 0.361	0.863	0.942
Soreness 4 period rolling CV (8 weeks)	108	9	0.207	-1.818 to 2.232	0.841	0.942

Note. Separate GEE models were fitted for each candidate feature using athlete ID as the clustering variable. FDR correction was applied across the 12 tests.

Supplementary Table S2. Univariable quantile-regression results at τ = 0.25, 0.50, and 0.75.

Feature	τ	β	95% CI	P value	FDR-adjusted P value
Training strain 4 period rolling mean (8 weeks)	0.25	-3.318×10^{-10}	-1.400×10^{-5} to 1.400×10^{-5}	1.000	1.000
Training strain 4 period rolling mean (8 weeks)	0.5	-4.968×10^{-11}	-1.417×10^{-5} to 1.417×10^{-5}	1.000	1.000
Training strain 4 period rolling mean (8 weeks)	0.75	-2.715×10^{-5}	-4.026×10^{-5} to -1.405×10^{-5}	0.001	0.002
Training strain 4 period rolling CV (8 weeks)	0.25	3.210×10^{-6}	-0.264 to 0.264	1.000	1.000
Training strain 4 period rolling CV (8 weeks)	0.5	1.519×10^{-6}	-0.195 to 0.195	1.000	1.000
Training strain 4 period rolling CV (8 weeks)	0.75	0.366	0.269 to 0.463	2.379×10^{-5}	1.345×10^{-4}
Training monotony 4 period rolling mean (8 weeks)	0.25	-7.353×10^{-6}	-0.452 to 0.452	1.000	1.000
Training monotony 4 period rolling mean (8 weeks)	0.5	-3.767×10^{-6}	-0.367 to 0.367	1.000	1.000
Training monotony 4 period rolling mean (8 weeks)	0.75	-0.868	-1.015 to -0.721	8.023×10^{-7}	9.627×10^{-6}
Training monotony 4 period rolling CV (8 weeks)	0.25	2.009×10^{-7}	-0.611 to 0.611	1.000	1.000
Training monotony 4 period rolling CV (8 weeks)	0.5	-4.324×10^{-6}	-0.319 to 0.319	1.000	1.000
Training monotony 4 period rolling CV (8 weeks)	0.75	0.934	0.664 to 1.205	4.485×10^{-5}	1.345×10^{-4}
Testosterone 4 period rolling mean (8 weeks)	0.25	5.259×10^{-4}	3.288×10^{-4} to 7.230×10^{-4}	2.735×10^{-4}	0.002
Testosterone 4 period rolling mean (8 weeks)	0.5	2.168×10^{-4}	-3.877×10^{-5} to 4.723×10^{-4}	0.086	0.148
Testosterone 4 period rolling mean (8 weeks)	0.75	9.883×10^{-5}	-2.167×10^{-4} to 4.144×10^{-4}	0.491	0.589
Testosterone 4 period rolling CV (8 weeks)	0.25	7.544×10^{-8}	-0.537 to 0.537	1.000	1.000
Testosterone 4 period rolling CV (8 weeks)	0.5	-0.660	-1.107 to -0.214	0.009	0.022
Testosterone 4 period rolling CV (8 weeks)	0.75	-1.453	-2.102 to -0.804	8.622×10^{-4}	0.001
CK 4 period rolling mean (8 weeks)	0.25	-8.315×10^{-4}	-0.001 to -4.288×10^{-4}	0.001	0.005

Feature	τ	β	95% CI	P value	FDR-adjusted P value
CK 4 period rolling mean (8 weeks)	0.5	-9.307×10^{-4}	-0.001 to -4.787×10^{-4}	0.001	0.004
CK 4 period rolling mean (8 weeks)	0.75	-9.572×10^{-11}	-6.925×10^{-4} to 6.925×10^{-4}	1.000	1.000
CK 4 period rolling CV (8 weeks)	0.25	0.535	0.460 to 0.610	1.858×10^{-7}	2.230×10^{-6}
CK 4 period rolling CV (8 weeks)	0.5	0.561	0.381 to 0.740	9.290×10^{-5}	6.443×10^{-4}
CK 4 period rolling CV (8 weeks)	0.75	0.665	0.403 to 0.928	3.880×10^{-4}	7.761×10^{-4}
Perceived sleep quality 4 period rolling mean (8 weeks)	0.25	0.163	-0.059 to 0.385	0.129	0.309
Perceived sleep quality 4 period rolling mean (8 weeks)	0.5	-0.166	-0.318 to -0.014	0.036	0.072
Perceived sleep quality 4 period rolling mean (8 weeks)	0.75	-9.035×10^{-7}	-0.161 to 0.161	1.000	1.000
Perceived sleep quality 4 period rolling CV (8 weeks)	0.25	1.597	0.814 to 2.379	0.002	0.005
Perceived sleep quality 4 period rolling CV (8 weeks)	0.5	2.199	1.436 to 2.962	1.611×10^{-4}	6.443×10^{-4}
Perceived sleep quality 4 period rolling CV (8 weeks)	0.75	2.135	1.531 to 2.739	3.806×10^{-5}	1.345×10^{-4}
Soreness 4 period rolling mean (8 weeks)	0.25	-0.079	-0.243 to 0.084	0.296	0.592
Soreness 4 period rolling mean (8 weeks)	0.5	0.245	0.161 to 0.329	1.527×10^{-4}	6.443×10^{-4}
Soreness 4 period rolling mean (8 weeks)	0.75	0.169	0.039 to 0.298	0.017	0.023
Soreness 4 period rolling CV (8 weeks)	0.25	-9.979×10^{-6}	-1.008 to 1.008	1.000	1.000
Soreness 4 period rolling CV (8 weeks)	0.5	-1.671×10^{-6}	-0.973 to 0.973	1.000	1.000
Soreness 4 period rolling CV (8 weeks)	0.75	1.737	1.148 to 2.327	1.382×10^{-4}	3.317×10^{-4}

Note. Each model included one candidate time-series feature. Robust covariance estimation was clustered by athlete ID, and FDR correction was applied separately within each quantile across the 12 candidate features.

Supplementary Table S3. Univariable quantile-regression results across τ = 0.10–0.90.

Feature	τ	β	95% CI	P value	FDR-adjusted P value
Training strain 4 period rolling mean (8 weeks)	0.1	-2.414×10^{-11}	[-2.536×10^{-5}, 2.536×10^{-5}]	1.000	1.000
Training strain 4 period rolling mean (8 weeks)	0.15	-6.177×10^{-11}	[-2.379×10^{-5}, 2.379×10^{-5}]	1.000	1.000
Training strain 4 period rolling mean (8 weeks)	0.2	-1.655×10^{-5}	[-2.683×10^{-5}, -6.271×10^{-6}]	0.006	0.024
Training strain 4 period rolling mean (8 weeks)	0.25	-3.318×10^{-10}	[-1.4×10^{-5}, 1.4×10^{-5}]	1.000	1.000
Training strain 4 period rolling mean (8 weeks)	0.3	-7.252×10^{-11}	[-1.421×10^{-5}, 1.421×10^{-5}]	1.000	1.000
Training strain 4 period rolling mean (8 weeks)	0.35	-1.276×10^{-10}	[-1.593×10^{-5}, 1.593×10^{-5}]	1.000	1.000
Training strain 4 period rolling mean (8 weeks)	0.4	-5.337×10^{-11}	[-1.599×10^{-5}, 1.599×10^{-5}]	1.000	1.000
Training strain 4 period rolling mean (8 weeks)	0.45	-1.345×10^{-5}	[-2.95×10^{-5}, 2.609×10^{-6}]	0.090	0.116
Training strain 4 period rolling mean (8 weeks)	0.5	-4.968×10^{-11}	[-1.417×10^{-5}, 1.417×10^{-5}]	1.000	1.000
Training strain 4 period rolling mean (8 weeks)	0.55	-3.143×10^{-10}	[-1.297×10^{-5}, 1.296×10^{-5}]	1.000	1.000
Training strain 4 period rolling mean (8 weeks)	0.6	-1.801×10^{-10}	[-1.763×10^{-5}, 1.763×10^{-5}]	1.000	1.000
Training strain 4 period rolling mean (8 weeks)	0.65	-1.284×10^{-5}	[-2.742×10^{-5}, 1.739×10^{-6}]	0.077	0.132
Training strain 4 period rolling mean (8 weeks)	0.7	-1.394×10^{-5}	[-2.704×10^{-5}, -8.364×10^{-7}]	0.040	0.060
Training strain 4 period rolling mean (8 weeks)	0.75	-2.715×10^{-5}	[-4.026×10^{-5}, -1.405×10^{-5}]	0.001	0.002
Training strain 4 period rolling mean (8 weeks)	0.8	-3.013×10^{-5}	[-3.865×10^{-5}, -2.16×10^{-5}]	3.819×10^{-5}	9.166×10^{-5}
Training strain 4 period rolling mean (8 weeks)	0.85	-3.558×10^{-10}	[-9.496×10^{-6}, 9.495×10^{-6}]	1.000	1.000
Training strain 4 period rolling mean (8 weeks)	0.9	-8.699×10^{-11}	[-1.844×10^{-5}, 1.844×10^{-5}]	1.000	1.000
Training strain 4 period rolling CV (8 weeks)	0.1	2.083×10^{-7}	[-0.606, 0.606]	1.000	1.000
Training strain 4 period rolling CV (8 weeks)	0.15	3.777×10^{-7}	[-0.576, 0.576]	1.000	1.000
Training strain 4 period rolling CV (8 weeks)	0.2	1.472×10^{-5}	[-0.553, 0.553]	1.000	1.000
Training strain 4 period rolling CV (8 weeks)	0.25	3.21×10^{-6}	[-0.264, 0.264]	1.000	1.000
Training strain 4 period rolling CV (8 weeks)	0.3	6.774×10^{-6}	[-0.257, 0.257]	1.000	1.000
Training strain 4 period rolling CV (8 weeks)	0.35	4.833×10^{-6}	[-0.289, 0.289]	1.000	1.000
Training strain 4 period rolling CV (8 weeks)	0.4	4.012×10^{-6}	[-0.287, 0.287]	1.000	1.000
Training strain 4 period rolling CV (8 weeks)	0.45	0.34	[0.076, 0.603]	0.018	0.030
Training strain 4 period rolling CV (8 weeks)	0.5	1.519×10^{-6}	[-0.195, 0.195]	1.000	1.000
Training strain 4 period rolling CV (8 weeks)	0.55	0.235	[0.049, 0.422]	0.020	0.026
Training strain 4 period rolling CV (8 weeks)	0.6	4.689×10^{-7}	[-0.174, 0.174]	1.000	1.000
Training strain 4 period rolling CV (8 weeks)	0.65	-2.638×10^{-7}	[-0.149, 0.149]	1.000	1.000
Training strain 4 period rolling CV (8 weeks)	0.7	2.882×10^{-5}	[-0.115, 0.115]	1.000	1.000
Training strain 4 period rolling CV (8 weeks)	0.75	0.366	[0.269, 0.463]	2.379×10^{-5}	1.345×10^{-4}
Training strain 4 period rolling CV (8 weeks)	0.8	0.547	[0.459, 0.636]	5.659×10^{-7}	3.395×10^{-6}

Feature	τ	β	95% CI	P value	FDR-adjusted P value
Training strain 4 period rolling CV (8 weeks)	0.85	2.402×10^{-6}	[-0.208, 0.208]	1.000	1.000
Training strain 4 period rolling CV (8 weeks)	0.9	8.475×10^{-7}	[-0.373, 0.373]	1.000	1.000
Training monotony 4 period rolling mean (8 weeks)	0.1	-6.82×10^{-7}	[-0.841, 0.841]	1.000	1.000
Training monotony 4 period rolling mean (8 weeks)	0.15	-1.586×10^{-6}	[-0.808, 0.808]	1.000	1.000
Training monotony 4 period rolling mean (8 weeks)	0.2	-0.551	[-1.023, -0.08]	0.027	0.082
Training monotony 4 period rolling mean (8 weeks)	0.25	-7.353×10^{-6}	[-0.452, 0.452]	1.000	1.000
Training monotony 4 period rolling mean (8 weeks)	0.3	-0.186	[-0.635, 0.264]	0.369	0.633
Training monotony 4 period rolling mean (8 weeks)	0.35	-0.33	[-0.851, 0.191]	0.182	0.546
Training monotony 4 period rolling mean (8 weeks)	0.4	-0.454	[-0.939, 0.032]	0.063	0.152
Training monotony 4 period rolling mean (8 weeks)	0.45	-0.389	[-0.865, 0.088]	0.097	0.116
Training monotony 4 period rolling mean (8 weeks)	0.5	-3.767×10^{-6}	[-0.367, 0.367]	1.000	1.000
Training monotony 4 period rolling mean (8 weeks)	0.55	-0.525	[-0.778, -0.272]	0.001	0.003
Training monotony 4 period rolling mean (8 weeks)	0.6	-0.847	[-1.07, -0.625]	2.221×10^{-5}	2.665×10^{-4}
Training monotony 4 period rolling mean (8 weeks)	0.65	-0.872	[-1.066, -0.678]	6.456×10^{-6}	7.748×10^{-5}
Training monotony 4 period rolling mean (8 weeks)	0.7	-0.809	[-0.963, -0.655]	1.982×10^{-6}	2.378×10^{-5}
Training monotony 4 period rolling mean (8 weeks)	0.75	-0.868	[-1.015, -0.721]	8.023×10^{-7}	9.627×10^{-6}
Training monotony 4 period rolling mean (8 weeks)	0.8	-0.832	[-0.924, -0.739]	3.11×10^{-8}	3.732×10^{-7}
Training monotony 4 period rolling mean (8 weeks)	0.85	-3.541×10^{-6}	[-0.235, 0.235]	1.000	1.000
Training monotony 4 period rolling mean (8 weeks)	0.9	-1.289×10^{-6}	[-0.368, 0.368]	1.000	1.000
Training monotony 4 period rolling CV (8 weeks)	0.1	6.022×10^{-8}	[-1.363, 1.363]	1.000	1.000
Training monotony 4 period rolling CV (8 weeks)	0.15	9.564×10^{-8}	[-1.282, 1.282]	1.000	1.000
Training monotony 4 period rolling CV (8 weeks)	0.2	3.12×10^{-6}	[-1.212, 1.212]	1.000	1.000
Training monotony 4 period rolling CV (8 weeks)	0.25	2.009×10^{-7}	[-0.611, 0.611]	1.000	1.000
Training monotony 4 period rolling CV (8 weeks)	0.3	-3.067×10^{-7}	[-0.587, 0.587]	1.000	1.000
Training monotony 4 period rolling CV (8 weeks)	0.35	-2.357×10^{-6}	[-0.573, 0.573]	1.000	1.000
Training monotony 4 period rolling CV (8 weeks)	0.4	-1.377×10^{-6}	[-0.571, 0.571]	1.000	1.000
Training monotony 4 period rolling CV (8 weeks)	0.45	-0.719	[-1.151, -0.287]	0.005	0.012
Training monotony 4 period rolling CV (8 weeks)	0.5	-4.324×10^{-6}	[-0.319, 0.319]	1.000	1.000
Training monotony 4 period rolling CV (8 weeks)	0.55	-0.762	[-1.125, -0.4]	0.001	0.003
Training monotony 4 period rolling CV (8 weeks)	0.6	-7.038×10^{-6}	[-0.229, 0.229]	1.000	1.000
Training monotony 4 period rolling CV (8 weeks)	0.65	-0.333	[-0.55, -0.117]	0.008	0.018
Training monotony 4 period rolling CV (8 weeks)	0.7	-0.334	[-0.504, -0.165]	0.002	0.004
Training monotony 4 period rolling CV (8 weeks)	0.75	0.934	[0.664, 1.205]	4.485×10^{-5}	1.345×10^{-4}

Feature	τ	β	95% CI	P value	FDR-adjusted P value
Training monotony 4 period rolling CV (8 weeks)	0.8	1.007	[0.787, 1.227]	5.61×10^{-6}	2.244×10^{-5}
Training monotony 4 period rolling CV (8 weeks)	0.85	1.956×10^{-6}	[-0.556, 0.556]	1.000	1.000
Training monotony 4 period rolling CV (8 weeks)	0.9	1.231×10^{-6}	[-0.607, 0.607]	1.000	1.000
Testosterone 4 period rolling mean (8 weeks)	0.1	1.605×10^{-8}	[-5.12×10^{-4}, 5.12×10^{-4}]	1.000	1.000
Testosterone 4 period rolling mean (8 weeks)	0.15	1.369×10^{-7}	[-4.165×10^{-4}, 4.168×10^{-4}]	0.999	1.000
Testosterone 4 period rolling mean (8 weeks)	0.2	4.623×10^{-4}	[2.881×10^{-4}, 6.365×10^{-4}]	2.832×10^{-4}	0.002
Testosterone 4 period rolling mean (8 weeks)	0.25	5.259×10^{-4}	[3.288×10^{-4}, 7.23×10^{-4}]	2.735×10^{-4}	0.002
Testosterone 4 period rolling mean (8 weeks)	0.3	5.448×10^{-4}	[3.156×10^{-4}, 7.741×10^{-4}]	5.874×10^{-4}	0.004
Testosterone 4 period rolling mean (8 weeks)	0.35	4.332×10^{-4}	[2.301×10^{-4}, 6.364×10^{-4}]	0.001	0.007
Testosterone 4 period rolling mean (8 weeks)	0.4	1.88×10^{-4}	[-4.271×10^{-5}, 4.187×10^{-4}]	0.097	0.194
Testosterone 4 period rolling mean (8 weeks)	0.45	1.901×10^{-4}	[-6.068×10^{-5}, 4.408×10^{-4}]	0.119	0.129
Testosterone 4 period rolling mean (8 weeks)	0.5	2.168×10^{-4}	[-3.877×10^{-5}, 4.723×10^{-4}]	0.086	0.148
Testosterone 4 period rolling mean (8 weeks)	0.55	4.437×10^{-4}	[2.173×10^{-4}, 6.7×10^{-4}]	0.002	0.004
Testosterone 4 period rolling mean (8 weeks)	0.6	9.109×10^{-5}	[-2.139×10^{-4}, 3.96×10^{-4}]	0.510	0.681
Testosterone 4 period rolling mean (8 weeks)	0.65	9.353×10^{-5}	[-2.117×10^{-4}, 3.987×10^{-4}]	0.500	0.600
Testosterone 4 period rolling mean (8 weeks)	0.7	9.815×10^{-5}	[-2.079×10^{-4}, 4.042×10^{-4}]	0.481	0.577
Testosterone 4 period rolling mean (8 weeks)	0.75	9.883×10^{-5}	[-2.167×10^{-4}, 4.144×10^{-4}]	0.491	0.589
Testosterone 4 period rolling mean (8 weeks)	0.8	1.135×10^{-8}	[-6.004×10^{-4}, 6.004×10^{-4}]	1.000	1.000
Testosterone 4 period rolling mean (8 weeks)	0.85	9.502×10^{-10}	[-6.32×10^{-4}, 6.32×10^{-4}]	1.000	1.000
Testosterone 4 period rolling mean (8 weeks)	0.9	7.492×10^{-11}	[-6.697×10^{-4}, 6.697×10^{-4}]	1.000	1.000
Testosterone 4 period rolling CV (8 weeks)	0.1	4.89×10^{-7}	[-0.831, 0.831]	1.000	1.000
Testosterone 4 period rolling CV (8 weeks)	0.15	1.19×10^{-6}	[-0.783, 0.783]	1.000	1.000
Testosterone 4 period rolling CV (8 weeks)	0.2	0.283	[-0.091, 0.657]	0.119	0.237
Testosterone 4 period rolling CV (8 weeks)	0.25	7.544×10^{-8}	[-0.537, 0.537]	1.000	1.000
Testosterone 4 period rolling CV (8 weeks)	0.3	-2.724×10^{-6}	[-0.426, 0.426]	1.000	1.000
Testosterone 4 period rolling CV (8 weeks)	0.35	-0.238	[-0.74, 0.265]	0.307	0.614
Testosterone 4 period rolling CV (8 weeks)	0.4	-0.397	[-0.8, 0.007]	0.053	0.152
Testosterone 4 period rolling CV (8 weeks)	0.45	-0.559	[-0.978, -0.14]	0.015	0.030
Testosterone 4 period rolling CV (8 weeks)	0.5	-0.66	[-1.107, -0.214]	0.009	0.022
Testosterone 4 period rolling CV (8 weeks)	0.55	-0.911	[-1.417, -0.404]	0.003	0.005
Testosterone 4 period rolling CV (8 weeks)	0.6	-0.963	[-1.418, -0.507]	0.001	0.004
Testosterone 4 period rolling CV (8 weeks)	0.65	-0.614	[-1.071, -0.157]	0.015	0.029
Testosterone 4 period rolling CV (8 weeks)	0.7	-0.675	[-1.144, -0.205]	0.011	0.018

Feature	τ	β	95% CI	P value	FDR-adjusted P value
Testosterone 4 period rolling CV (8 weeks)	0.75	-1.453	[-2.102, -0.804]	8.622×10^{-4}	0.001
Testosterone 4 period rolling CV (8 weeks)	0.8	-0.91	[-1.657, -0.162]	0.023	0.039
Testosterone 4 period rolling CV (8 weeks)	0.85	-0.963	[-1.86, -0.067]	0.038	0.459
Testosterone 4 period rolling CV (8 weeks)	0.9	-5.336×10^{-5}	[-0.11, 0.11]	0.999	1.000
CK 4 period rolling mean (8 weeks)	0.1	-5.7×10^{-10}	[-9.757×10^{-4}, 9.757×10^{-4}]	1.000	1.000
CK 4 period rolling mean (8 weeks)	0.15	-1.812×10^{-9}	[-9.52×10^{-4}, 9.52×10^{-4}]	1.000	1.000
CK 4 period rolling mean (8 weeks)	0.2	-4.074×10^{-8}	[-7.496×10^{-4}, 7.495×10^{-4}]	1.000	1.000
CK 4 period rolling mean (8 weeks)	0.25	-8.315×10^{-4}	[-0.001, -4.288×10^{-4}]	0.001	0.005
CK 4 period rolling mean (8 weeks)	0.3	-4.498×10^{-4}	[-8.399×10^{-4}, -5.965×10^{-5}]	0.029	0.087
CK 4 period rolling mean (8 weeks)	0.35	-4.075×10^{-4}	[-8.337×10^{-4}, 1.876×10^{-5}]	0.059	0.234
CK 4 period rolling mean (8 weeks)	0.4	-8.804×10^{-4}	[-0.001, -5.225×10^{-4}]	4.688×10^{-4}	0.003
CK 4 period rolling mean (8 weeks)	0.45	-7.5×10^{-4}	[-0.001, -3.479×10^{-4}]	0.003	0.008
CK 4 period rolling mean (8 weeks)	0.5	-9.307×10^{-4}	[-0.001, -4.787×10^{-4}]	0.001	0.004
CK 4 period rolling mean (8 weeks)	0.55	-9.057×10^{-4}	[-0.001, -4.316×10^{-4}]	0.002	0.004
CK 4 period rolling mean (8 weeks)	0.6	-8.445×10^{-4}	[-0.001, -4.207×10^{-4}]	0.002	0.004
CK 4 period rolling mean (8 weeks)	0.65	1.226×10^{-9}	[-7.826×10^{-4}, 7.826×10^{-4}]	1.000	1.000
CK 4 period rolling mean (8 weeks)	0.7	1.577×10^{-9}	[-7.251×10^{-4}, 7.251×10^{-4}]	1.000	1.000
CK 4 period rolling mean (8 weeks)	0.75	-9.572×10^{-11}	[-6.925×10^{-4}, 6.925×10^{-4}]	1.000	1.000
CK 4 period rolling mean (8 weeks)	0.8	-6×10^{-8}	[-0.001, 0.001]	1.000	1.000
CK 4 period rolling mean (8 weeks)	0.85	-1.671×10^{-8}	[-9.785×10^{-4}, 9.784×10^{-4}]	1.000	1.000
CK 4 period rolling mean (8 weeks)	0.9	-2.519×10^{-10}	[-9.531×10^{-4}, 9.531×10^{-4}]	1.000	1.000
CK 4 period rolling CV (8 weeks)	0.1	0.662	[0.512, 0.811]	7.171×10^{-6}	8.605×10^{-5}
CK 4 period rolling CV (8 weeks)	0.15	0.65	[0.553, 0.746]	3.009×10^{-7}	3.611×10^{-6}
CK 4 period rolling CV (8 weeks)	0.2	0.638	[0.499, 0.778]	5.713×10^{-6}	6.856×10^{-5}
CK 4 period rolling CV (8 weeks)	0.25	0.535	[0.46, 0.61]	1.858×10^{-7}	2.23×10^{-6}
CK 4 period rolling CV (8 weeks)	0.3	0.489	[0.399, 0.58]	1.595×10^{-6}	1.914×10^{-5}
CK 4 period rolling CV (8 weeks)	0.35	0.459	[0.369, 0.548]	2.491×10^{-6}	2.99×10^{-5}
CK 4 period rolling CV (8 weeks)	0.4	0.448	[0.343, 0.553]	9.481×10^{-6}	1.138×10^{-4}
CK 4 period rolling CV (8 weeks)	0.45	0.426	[0.328, 0.525]	8.562×10^{-6}	1.027×10^{-4}
CK 4 period rolling CV (8 weeks)	0.5	0.561	[0.381, 0.74]	9.29×10^{-5}	6.443×10^{-4}
CK 4 period rolling CV (8 weeks)	0.55	0.358	[0.227, 0.489]	2.359×10^{-4}	0.001
CK 4 period rolling CV (8 weeks)	0.6	0.328	[0.206, 0.449]	2.568×10^{-4}	0.001
CK 4 period rolling CV (8 weeks)	0.65	0.614	[0.344, 0.885]	7.791×10^{-4}	0.003

Feature	τ	β	95% CI	P value	FDR-adjusted P value
CK 4 period rolling CV (8 weeks)	0.7	0.584	[0.328, 0.839]	7.603×10^{-4}	0.002
CK 4 period rolling CV (8 weeks)	0.75	0.665	[0.403, 0.928]	3.88×10^{-4}	7.761×10^{-4}
CK 4 period rolling CV (8 weeks)	0.8	0.609	[0.414, 0.804]	9.195×10^{-5}	1.839×10^{-4}
CK 4 period rolling CV (8 weeks)	0.85	1.538×10^{-6}	[-0.136, 0.136]	1.000	1.000
CK 4 period rolling CV (8 weeks)	0.9	9.068×10^{-7}	[-0.123, 0.123]	1.000	1.000
Perceived sleep quality 4 period rolling mean (8 weeks)	0.1	2.521×10^{-7}	[-0.391, 0.391]	1.000	1.000
Perceived sleep quality 4 period rolling mean (8 weeks)	0.15	4.168×10^{-7}	[-0.375, 0.375]	1.000	1.000
Perceived sleep quality 4 period rolling mean (8 weeks)	0.2	0.157	[0.008, 0.307]	0.041	0.098
Perceived sleep quality 4 period rolling mean (8 weeks)	0.25	0.163	[-0.059, 0.385]	0.129	0.309
Perceived sleep quality 4 period rolling mean (8 weeks)	0.3	-1.144×10^{-6}	[-0.191, 0.191]	1.000	1.000
Perceived sleep quality 4 period rolling mean (8 weeks)	0.35	-0.067	[-0.293, 0.16]	0.517	0.775
Perceived sleep quality 4 period rolling mean (8 weeks)	0.4	-0.206	[-0.358, -0.055]	0.014	0.055
Perceived sleep quality 4 period rolling mean (8 weeks)	0.45	-0.186	[-0.339, -0.033]	0.023	0.034
Perceived sleep quality 4 period rolling mean (8 weeks)	0.5	-0.166	[-0.318, -0.014]	0.036	0.072
Perceived sleep quality 4 period rolling mean (8 weeks)	0.55	-0.192	[-0.35, -0.034]	0.023	0.027
Perceived sleep quality 4 period rolling mean (8 weeks)	0.6	-0.085	[-0.288, 0.119]	0.367	0.550
Perceived sleep quality 4 period rolling mean (8 weeks)	0.65	-0.074	[-0.279, 0.131]	0.428	0.571
Perceived sleep quality 4 period rolling mean (8 weeks)	0.7	-0.072	[-0.267, 0.123]	0.421	0.561
Perceived sleep quality 4 period rolling mean (8 weeks)	0.75	-9.035×10^{-7}	[-0.161, 0.161]	1.000	1.000
Perceived sleep quality 4 period rolling mean (8 weeks)	0.8	2.465×10^{-6}	[-0.378, 0.378]	1.000	1.000
Perceived sleep quality 4 period rolling mean (8 weeks)	0.85	1.494×10^{-7}	[-0.393, 0.393]	1.000	1.000
Perceived sleep quality 4 period rolling mean (8 weeks)	0.9	9.275×10^{-8}	[-0.412, 0.412]	1.000	1.000
Perceived sleep quality 4 period rolling CV (8 weeks)	0.1	1.179×10^{-6}	[-2.141, 2.141]	1.000	1.000
Perceived sleep quality 4 period rolling CV (8 weeks)	0.15	1.872×10^{-6}	[-2.173, 2.173]	1.000	1.000
Perceived sleep quality 4 period rolling CV (8 weeks)	0.2	4.928×10^{-6}	[-2.281, 2.281]	1.000	1.000
Perceived sleep quality 4 period rolling CV (8 weeks)	0.25	1.597	[0.814, 2.379]	0.002	0.005
Perceived sleep quality 4 period rolling CV (8 weeks)	0.3	1.379	[0.475, 2.284]	0.008	0.032
Perceived sleep quality 4 period rolling CV (8 weeks)	0.35	2.838×10^{-5}	[-1.608, 1.608]	1.000	1.000
Perceived sleep quality 4 period rolling CV (8 weeks)	0.4	1.338×10^{-5}	[-1.719, 1.719]	1.000	1.000
Perceived sleep quality 4 period rolling CV (8 weeks)	0.45	2.476	[1.603, 3.349]	1.8×10^{-4}	7.201×10^{-4}
Perceived sleep quality 4 period rolling CV (8 weeks)	0.5	2.199	[1.436, 2.962]	1.611×10^{-4}	6.443×10^{-4}
Perceived sleep quality 4 period rolling CV (8 weeks)	0.55	1.94	[1.195, 2.685]	3.213×10^{-4}	0.001
Perceived sleep quality 4 period rolling CV (8 weeks)	0.6	1.338	[0.558, 2.117]	0.004	0.008

Feature	τ	β	95% CI	P value	FDR-adjusted P value
Perceived sleep quality 4 period rolling CV (8 weeks)	0.65	1.245	[0.691, 1.798]	8.365×10^{-4}	0.003
Perceived sleep quality 4 period rolling CV (8 weeks)	0.7	1.291	[0.8, 1.782]	3.006×10^{-4}	0.002
Perceived sleep quality 4 period rolling CV (8 weeks)	0.75	2.135	[1.531, 2.739]	3.806×10^{-5}	1.345×10^{-4}
Perceived sleep quality 4 period rolling CV (8 weeks)	0.8	3.162×10^{-4}	[-0.888, 0.889]	0.999	1.000
Perceived sleep quality 4 period rolling CV (8 weeks)	0.85	4.623×10^{-6}	[-1.428, 1.428]	1.000	1.000
Perceived sleep quality 4 period rolling CV (8 weeks)	0.9	3.01×10^{-6}	[-1.523, 1.523]	1.000	1.000
Soreness 4 period rolling mean (8 weeks)	0.1	-2.292×10^{-7}	[-0.151, 0.151]	1.000	1.000
Soreness 4 period rolling mean (8 weeks)	0.15	-8.882×10^{-7}	[-0.106, 0.106]	1.000	1.000
Soreness 4 period rolling mean (8 weeks)	0.2	-0.078	[-0.202, 0.046]	0.186	0.319
Soreness 4 period rolling mean (8 weeks)	0.25	-0.079	[-0.243, 0.084]	0.296	0.592
Soreness 4 period rolling mean (8 weeks)	0.3	-0.095	[-0.28, 0.09]	0.270	0.540
Soreness 4 period rolling mean (8 weeks)	0.35	-0.108	[-0.321, 0.104]	0.274	0.614
Soreness 4 period rolling mean (8 weeks)	0.4	1.387×10^{-6}	[-0.214, 0.214]	1.000	1.000
Soreness 4 period rolling mean (8 weeks)	0.45	0.259	[0.172, 0.346]	1.245×10^{-4}	7.201×10^{-4}
Soreness 4 period rolling mean (8 weeks)	0.5	0.245	[0.161, 0.329]	1.527×10^{-4}	6.443×10^{-4}
Soreness 4 period rolling mean (8 weeks)	0.55	0.249	[0.168, 0.329]	9.769×10^{-5}	0.001
Soreness 4 period rolling mean (8 weeks)	0.6	0.239	[0.158, 0.32]	1.377×10^{-4}	8.262×10^{-4}
Soreness 4 period rolling mean (8 weeks)	0.65	0.242	[0.168, 0.315]	6.64×10^{-5}	3.984×10^{-4}
Soreness 4 period rolling mean (8 weeks)	0.7	0.207	[0.117, 0.298]	7.284×10^{-4}	0.002
Soreness 4 period rolling mean (8 weeks)	0.75	0.169	[0.039, 0.298]	0.017	0.023
Soreness 4 period rolling mean (8 weeks)	0.8	0.137	[0.004, 0.271]	0.045	0.068
Soreness 4 period rolling mean (8 weeks)	0.85	1.742×10^{-6}	[-0.392, 0.392]	1.000	1.000
Soreness 4 period rolling mean (8 weeks)	0.9	2.05×10^{-7}	[-0.536, 0.536]	1.000	1.000
Soreness 4 period rolling CV (8 weeks)	0.1	-6.231×10^{-7}	[-2.314, 2.314]	1.000	1.000
Soreness 4 period rolling CV (8 weeks)	0.15	-9.897×10^{-7}	[-2.259, 2.259]	1.000	1.000
Soreness 4 period rolling CV (8 weeks)	0.2	-2.199×10^{-6}	[-2.253, 2.253]	1.000	1.000
Soreness 4 period rolling CV (8 weeks)	0.25	-9.979×10^{-6}	[-1.008, 1.008]	1.000	1.000
Soreness 4 period rolling CV (8 weeks)	0.3	-0.644	[-1.838, 0.55]	0.249	0.540
Soreness 4 period rolling CV (8 weeks)	0.35	-0.483	[-1.896, 0.93]	0.453	0.775
Soreness 4 period rolling CV (8 weeks)	0.4	-5.816×10^{-6}	[-1.434, 1.434]	1.000	1.000
Soreness 4 period rolling CV (8 weeks)	0.45	6.751×10^{-6}	[-0.934, 0.934]	1.000	1.000
Soreness 4 period rolling CV (8 weeks)	0.5	-1.671×10^{-6}	[-0.973, 0.973]	1.000	1.000
Soreness 4 period rolling CV (8 weeks)	0.55	-1.043	[-2.186, 0.1]	0.069	0.075

Feature	τ	β	95% CI	P value	FDR-adjusted P value
Soreness 4 period rolling CV (8 weeks)	0.6	0.625	[-0.084, 1.334]	0.077	0.131
Soreness 4 period rolling CV (8 weeks)	0.65	0.524	[-0.119, 1.167]	0.097	0.146
Soreness 4 period rolling CV (8 weeks)	0.7	0.835	[0.292, 1.377]	0.008	0.015
Soreness 4 period rolling CV (8 weeks)	0.75	1.737	[1.148, 2.327]	$1.382{\times}10^{-4}$	$3.317{\times}10^{-4}$
Soreness 4 period rolling CV (8 weeks)	0.8	1.567	[1.168, 1.966]	$1.768{\times}10^{-5}$	$5.303{\times}10^{-5}$
Soreness 4 period rolling CV (8 weeks)	0.85	$3.718{\times}10^{-6}$	[-1.494, 1.494]	1.000	1.000
Soreness 4 period rolling CV (8 weeks)	0.9	$2.341{\times}10^{-6}$	[-1.61, 1.61]	1.000	1.000

Note. Each model included one candidate time-series feature. Robust covariance estimation was clustered by athlete ID (9 athletes; 108 rolling observations). FDR correction was applied separately within each quantile across the 12 candidate features. Quantiles were evaluated from τ = 0.10 to 0.90 in 0.05 increments.

Supplementary Table S4. Six-predictor main quantile-regression model at τ = 0.25, 0.50, and 0.75.

Feature	τ	n	No. of athletes	β	95% CI	P value	FDR-adjusted P value
Training strain 4 period rolling mean (8 weeks)	0.25	108	9	5.121×10^{-5}	2.835×10^{-5} to 7.407×10^{-5}	8.574×10^{-4}	0.001
Training monotony 4 period rolling mean (8 weeks)	0.25	108	9	-1.351	-1.728 to -0.975	3.385×10^{-5}	6.771×10^{-5}
Testosterone 4 period rolling mean (8 weeks)	0.25	108	9	4.386×10^{-4}	3.264×10^{-4} to 5.508×10^{-4}	1.830×10^{-5}	5.490×10^{-5}
CK 4 period rolling mean (8 weeks)	0.25	108	9	-7.243×10^{-4}	-8.302×10^{-4} to -6.183×10^{-4}	2.621×10^{-7}	1.572×10^{-6}
Perceived sleep quality 4 period rolling CV (8 weeks)	0.25	108	9	1.748	1.017 to 2.479	5.630×10^{-4}	8.444×10^{-4}
Soreness 4 period rolling mean (8 weeks)	0.25	108	9	-0.162	-0.267 to -0.057	0.007	0.007
Training strain 4 period rolling mean (8 weeks)	0.5	108	9	2.861×10^{-5}	1.139×10^{-5} to 4.583×10^{-5}	0.005	0.005
Training monotony 4 period rolling mean (8 weeks)	0.5	108	9	-1.516	-1.876 to -1.155	1.075×10^{-5}	6.448×10^{-5}
Testosterone 4 period rolling mean (8 weeks)	0.5	108	9	4.427×10^{-4}	3.193×10^{-4} to 5.660×10^{-4}	3.421×10^{-5}	6.842×10^{-5}
CK 4 period rolling mean (8 weeks)	0.5	108	9	-7.823×10^{-4}	-0.001 to -5.043×10^{-4}	1.902×10^{-4}	2.853×10^{-4}
Perceived sleep quality 4 period rolling CV (8 weeks)	0.5	108	9	1.972	1.427 to 2.516	3.192×10^{-5}	6.842×10^{-5}
Soreness 4 period rolling mean (8 weeks)	0.5	108	9	0.152	0.071 to 0.234	0.003	0.003
Training strain 4 period rolling mean (8 weeks)	0.75	108	9	1.203×10^{-5}	-6.425×10^{-6} to 3.049×10^{-5}	0.171	0.171
Training monotony 4 period rolling mean (8 weeks)	0.75	108	9	-0.886	-1.290 to -0.483	9.727×10^{-4}	0.001
Testosterone 4 period rolling mean (8 weeks)	0.75	108	9	6.039×10^{-4}	4.770×10^{-4} to 7.308×10^{-4}	4.221×10^{-6}	2.416×10^{-5}
CK 4 period rolling mean (8 weeks)	0.75	108	9	-0.001	-0.001 to -8.262×10^{-4}	8.052×10^{-6}	2.416×10^{-5}
Perceived sleep quality 4 period rolling CV (8 weeks)	0.75	108	9	1.587	1.071 to 2.103	1.023×10^{-4}	1.616×10^{-4}
Soreness 4 period rolling mean (8 weeks)	0.75	108	9	0.227	0.152 to 0.301	1.077×10^{-4}	1.616×10^{-4}

Note. The main model included training strain rolling mean, training monotony rolling mean, testosterone rolling mean, CK rolling mean, perceived sleep-quality rolling CV, and soreness rolling mean. FDR correction was applied separately within each quantile across the six predictors.

Supplementary Table S5. Twelve-feature extended quantile-regression model at τ = 0.25, 0.50, and 0.75.

Feature	τ	β	95% CI	P value	FDR-adjusted P value
Training strain 4period rolling mean (8 weeks)	0.25	2.894×10^{-5}	1.253×10^{-5} to 4.535×10^{-5}	0.004	0.004
Training strain 4period rolling CV (8 weeks)	0.25	-0.140	-0.220 to -0.059	0.004	0.004
Training monotony 4period rolling mean (8 weeks)	0.25	-0.826	-1.061 to -0.591	3.953×10^{-5}	7.907×10^{-5}
Training monotony 4period rolling CV (8 weeks)	0.25	-0.721	-1.006 to -0.436	3.897×10^{-4}	6.680×10^{-4}
Testosterone 4period rolling mean (8 weeks)	0.25	4.188×10^{-4}	3.661×10^{-4} to 4.716×10^{-4}	8.144×10^{-8}	3.258×10^{-7}
Testosterone 4period rolling CV (8 weeks)	0.25	0.029	-0.163 to 0.220	0.737	0.737
CK 4period rolling mean (8 weeks)	0.25	-0.001	-0.001 to -0.001	7.328×10^{-10}	4.397×10^{-9}
CK 4 period rolling CV (8 weeks)	0.25	0.714	0.682 to 0.746	2.156×10^{-11}	2.587×10^{-10}
Perceived sleep quality 4 period rolling mean (8 weeks)	0.25	0.152	0.116 to 0.188	1.074×10^{-5}	2.577×10^{-5}
Perceived sleep quality 4 period rolling CV (8 weeks)	0.25	2.239	1.798 to 2.679	2.556×10^{-6}	7.668×10^{-6}
Soreness 4 period rolling mean (8 weeks)	0.25	-0.142	-0.209 to -0.075	0.001	0.002
Soreness 4 period rolling CV (8 weeks)	0.25	-0.239	-0.372 to -0.107	0.003	0.004
Training strain 4 period rolling mean (8 weeks)	0.5	8.232×10^{-7}	-5.638×10^{-6} to 7.284×10^{-6}	0.776	0.776
Training strain 4 period rolling CV (8 weeks)	0.5	0.061	0.027 to 0.096	0.004	0.004
Training monotony 4 period rolling mean (8 weeks)	0.5	-0.835	-0.934 to -0.736	5.199×10^{-8}	1.040×10^{-7}
Training monotony 4 period rolling CV (8 weeks)	0.5	-1.052	-1.221 to -0.883	5.449×10^{-7}	8.966×10^{-7}
Testosterone 4 period rolling mean (8 weeks)	0.5	4.649×10^{-4}	4.314×10^{-4} to 4.983×10^{-4}	9.747×10^{-10}	3.899×10^{-9}
Testosterone 4 period rolling CV (8 weeks)	0.5	0.327	0.261 to 0.392	3.103×10^{-6}	4.138×10^{-6}

Feature	τ	β	95% CI	P value	FDR-adjusted P value
CK 4 period rolling mean (8 weeks)	0.5	-0.002	-0.002 to -0.001	4.153×10^{-11}	3.452×10^{-10}
CK 4 period rolling CV (8 weeks)	0.5	0.788	0.748 to 0.828	5.754×10^{-11}	3.452×10^{-10}
Perceived sleep quality 4 period rolling mean (8 weeks)	0.5	0.335	0.307 to 0.363	3.352×10^{-9}	1.006×10^{-8}
Perceived sleep quality 4 period rolling CV (8 weeks)	0.5	2.266	2.065 to 2.468	5.274×10^{-9}	1.266×10^{-8}
Soreness 4 period rolling mean (8 weeks)	0.5	0.125	0.092 to 0.158	2.402×10^{-5}	2.882×10^{-5}
Soreness 4 period rolling CV (8 weeks)	0.5	-0.539	-0.627 to -0.451	5.977×10^{-7}	8.966×10^{-7}
Training strain 4 period rolling mean (8 weeks)	0.75	3.771×10^{-5}	3.090×10^{-5} to 4.452×10^{-5}	1.337×10^{-6}	2.292×10^{-6}
Training strain 4 period rolling CV (8 weeks)	0.75	0.383	0.299 to 0.467	5.865×10^{-6}	8.797×10^{-6}
Training monotony 4 period rolling mean (8 weeks)	0.75	-1.886	-2.051 to -1.720	4.745×10^{-9}	4.777×10^{-8}
Training monotony 4 period rolling CV (8 weeks)	0.75	-1.380	-1.688 to -1.072	6.664×10^{-6}	8.886×10^{-6}
Testosterone 4 period rolling mean (8 weeks)	0.75	5.194×10^{-4}	4.701×10^{-4} to 5.688×10^{-4}	8.860×10^{-9}	4.777×10^{-8}
Testosterone 4 period rolling CV (8 weeks)	0.75	-0.140	-0.263 to -0.017	0.031	0.031
CK 4 period rolling mean (8 weeks)	0.75	-9.956×10^{-4}	-0.001 to -8.974×10^{-4}	1.194×10^{-8}	4.777×10^{-8}
CK 4 period rolling CV (8 weeks)	0.75	0.313	0.240 to 0.387	9.795×10^{-6}	1.175×10^{-5}
Perceived sleep quality 4 period rolling mean (8 weeks)	0.75	0.287	0.254 to 0.321	4.542×10^{-8}	1.363×10^{-7}
Perceived sleep quality 4 period rolling CV (8 weeks)	0.75	1.910	1.678 to 2.143	6.292×10^{-8}	1.510×10^{-7}
Soreness 4 period rolling mean (8 weeks)	0.75	0.213	0.182 to 0.243	2.215×10^{-7}	4.429×10^{-7}
Soreness 4 period rolling CV (8 weeks)	0.75	0.306	0.153 to 0.460	0.002	0.002

Note. The extended model included all 12 candidate time-series features and was treated as an exploratory sensitivity analysis. FDR correction was applied separately within each quantile across the 12 predictors.

Supplementary Table S6. Variance inflation factors for the six-predictor main model.

Feature	VIF
Training strain 4 period rolling mean (8 weeks)	7.314
Training monotony 4 period rolling mean (8 weeks)	6.709
Testosterone 4 period rolling mean (8 weeks)	1.481
CK 4 period rolling mean (8 weeks)	1.276
Perceived sleep quality 4 period rolling CV (8 weeks)	1.084
Soreness 4 period rolling mean (8 weeks)	1.831

Supplementary Table S7. Variance inflation factors for the 12-feature extended model.

Feature	VIF
Training strain 4 period rolling mean (8 weeks)	9.425
Training strain 4 period rolling CV (8 weeks)	7.724
Training monotony 4 period rolling mean (8 weeks)	9.536
Training monotony 4 period rolling CV (8 weeks)	8.215
Testosterone 4 period rolling mean (8 weeks)	2.448
Testosterone 4 period rolling CV (8 weeks)	2.743
CK 4 period rolling mean (8 weeks)	1.762
CK 4 period rolling CV (8 weeks)	1.477
Perceived sleep quality 4 period rolling mean (8 weeks)	1.500
Perceived sleep quality 4 period rolling CV (8 weeks)	1.159
Soreness 4 period rolling mean (8 weeks)	2.319
Soreness 4 period rolling CV (8 weeks)	1.422

Supplementary Table S8. Small-cluster bias-reduced GEE results for the 12 candidate time-series features.

Feature	β	Bias-reduced 95% CI	P value	FDR-adjusted P value
Training strain rolling mean	-9.889×10^{-6}	-4.904×10^{-5} to 2.926×10^{-5}	0.621	0.964
Training strain rolling CV	0.156	-0.343 to 0.656	0.540	0.964
Training monotony rolling mean	-0.381	-1.310 to 0.547	0.421	0.964
Training monotony rolling CV	0.019	-0.811 to 0.849	0.964	0.964
Testosterone rolling mean	1.651×10^{-4}	-5.579×10^{-4} to 8.882×10^{-4}	0.654	0.964
Testosterone rolling CV	-0.473	-1.737 to 0.791	0.463	0.964
CK rolling mean	-5.291×10^{-4}	-0.002 to 9.774×10^{-4}	0.491	0.964
CK rolling CV	0.529	-0.814 to 1.872	0.440	0.964
Perceived sleep quality rolling mean	-0.036	-0.552 to 0.479	0.890	0.964
Perceived sleep quality rolling CV	1.201	-2.902 to 5.303	0.566	0.964
Soreness rolling mean	0.029	-0.548 to 0.607	0.921	0.964
Soreness rolling CV	0.207	-2.483 to 2.897	0.880	0.964

Note. Bias-reduced covariance estimation was used to assess the sensitivity of GEE inference to the small number of athlete clusters.

Supplementary Table S9. GEE working correlation structures and convergence diagnostics.

Feature	Working correlation structure	Converged	β	Estimated correlation parameter	Note
Training strain rolling mean	Independence	Yes	-9.889×10^{-6}		
Training strain rolling mean	Exchangeable	Yes	-1.515×10^{-8}	0.985	
Training strain rolling mean	AR(1)	No			AR(1) correlation parameter could not be stably estimated
Training strain rolling CV	Independence	Yes	0.156		
Training strain rolling CV	Exchangeable	Yes	2.185×10^{-4}	0.985	
Training strain rolling CV	AR(1)	No			AR(1) correlation parameter could not be stably estimated
Training monotony rolling mean	Independence	Yes	-0.381		
Training monotony rolling mean	Exchangeable	Yes	-5.582×10^{-4}	0.985	
Training monotony rolling mean	AR(1)	No			AR(1) correlation parameter could not be stably estimated
Training monotony rolling CV	Independence	Yes	0.019		
Training monotony rolling CV	Exchangeable	Yes	2.676×10^{-5}	0.985	
Training monotony rolling CV	AR(1)	No			AR(1) correlation parameter could not be stably estimated
Testosterone rolling mean	Independence	Yes	1.651×10^{-4}		
Testosterone rolling mean	Exchangeable	Yes	9.841×10^{-6}	0.985	
Testosterone rolling mean	AR(1)	No			AR(1) correlation parameter could not be stably estimated
Testosterone rolling CV	Independence	Yes	-0.473		
Testosterone rolling CV	Exchangeable	Yes	-9.210×10^{-4}	0.985	
Testosterone rolling CV	AR(1)	No			AR(1) correlation parameter could not be stably estimated
CK rolling mean	Independence	Yes	-5.291×10^{-4}		

Feature	Working correlation structure	Converged	β	Estimated correlation parameter	Note
CK rolling mean	Exchangeable	Yes	-2.029×10^{-6}	0.985	
CK rolling mean	AR(1)	No			AR(1) correlation parameter could not be stably estimated
CK rolling CV	Independence	Yes	0.529		
CK rolling CV	Exchangeable	Yes	9.759×10^{-4}	0.985	
CK rolling CV	AR(1)	No			AR(1) correlation parameter could not be stably estimated
Perceived sleep quality rolling mean	Independence	Yes	-0.036		
Perceived sleep quality rolling mean	Exchangeable	Yes	-1.336×10^{-4}	0.985	
Perceived sleep quality rolling mean	AR(1)	No			AR(1) correlation parameter could not be stably estimated
Perceived sleep quality rolling CV	Independence	Yes	1.201		
Perceived sleep quality rolling CV	Exchangeable	Yes	0.002	0.985	
Perceived sleep quality rolling CV	AR(1)	No			AR(1) correlation parameter could not be stably estimated
Soreness rolling mean	Independence	Yes	0.029		
Soreness rolling mean	Exchangeable	Yes	1.674×10^{-4}	0.985	
Soreness rolling mean	AR(1)	No			AR(1) correlation parameter could not be stably estimated
Soreness rolling CV	Independence	Yes	0.207		
Soreness rolling CV	Exchangeable	Yes	3.328×10^{-4}	0.985	
Soreness rolling CV	AR(1)	No			AR(1) correlation parameter could not be stably estimated

Note. Independence, exchangeable, and AR(1) working correlation structures were examined. AR(1) did not yield stable estimates.

Supplementary Table S10. Leave-one-athlete-out analysis for the GEE models.

Feature	Full-sample β	Minimum LOAO β	Maximum LOAO β	No. retaining same direction
Training strain rolling mean	-9.889×10^{-6}	-1.799×10^{-5}	4.701×10^{-6}	8/9
Training strain rolling CV	0.156	-0.043	0.278	8/9
Training monotony rolling mean	-0.381	-0.505	0.031	8/9
Training monotony rolling CV	0.019	-0.276	0.290	6/9
Testosterone rolling mean	1.651×10^{-4}	-2.717×10^{-5}	3.797×10^{-4}	8/9
Testosterone rolling CV	-0.473	-1.008	-0.201	9/9
CK rolling mean	-5.291×10^{-4}	-8.555×10^{-4}	-1.082×10^{-4}	9/9
CK rolling CV	0.529	0.388	1.195	9/9
Perceived sleep quality rolling mean	-0.036	-0.198	0.058	5/9
Perceived sleep quality rolling CV	1.201	-0.732	1.793	8/9
Soreness rolling mean	0.029	-0.177	0.223	6/9
Soreness rolling CV	0.207	-0.965	0.691	8/9

Note. Each analysis sequentially excluded one athlete and refitted the corresponding GEE model.

Supplementary Table S11. Leave-one-athlete-out analysis for the six-predictor main quantile-regression model.

τ	Predictor	Full-sample β	Minimum LOAO β	Maximum LOAO β	No. retaining same direction
0.25	Training strain rolling mean	5.121×10^{-5}	-3.056×10^{-5}	7.003×10^{-5}	8/9
0.25	Training monotony rolling mean	-1.351	-1.966	0.383	8/9
0.25	Testosterone rolling mean	4.386×10^{-4}	1.249×10^{-4}	5.986×10^{-4}	9/9
0.25	CK rolling mean	-7.243×10^{-4}	-0.003	-1.447×10^{-4}	9/9
0.25	Perceived sleep quality rolling CV	1.748	-1.484	2.975	8/9
0.25	Soreness rolling mean	-0.162	-0.247	0.348	7/9
0.5	Training strain rolling mean	2.861×10^{-5}	-3.703×10^{-5}	9.287×10^{-5}	6/9
0.5	Training monotony rolling mean	-1.516	-2.405	-0.123	9/9
0.5	Testosterone rolling mean	4.427×10^{-4}	-9.420×10^{-6}	6.376×10^{-4}	8/9
0.5	CK rolling mean	-7.823×10^{-4}	-0.002	-1.978×10^{-4}	9/9
0.5	Perceived sleep quality rolling CV	1.972	-1.935	3.833	8/9
0.5	Soreness rolling mean	0.152	-0.375	0.261	8/9
0.75	Training strain rolling mean	1.203×10^{-5}	-3.198×10^{-5}	9.171×10^{-5}	8/9
0.75	Training monotony rolling mean	-0.886	-2.337	-0.153	9/9
0.75	Testosterone rolling mean	6.039×10^{-4}	2.239×10^{-4}	7.524×10^{-4}	9/9
0.75	CK rolling mean	-0.001	-0.001	-5.102×10^{-4}	9/9
0.75	Perceived sleep quality rolling CV	1.587	-0.290	2.380	8/9
0.75	Soreness rolling mean	0.227	-0.311	0.338	8/9

Note. Each analysis sequentially excluded one athlete and refitted the six-predictor main model at τ = 0.25, 0.50, and 0.75.

Supplementary Table S12. Log10-scale sensitivity analysis for the GEE models.

Feature	Original skewness	Scale	Post-transformation skewness	β	Conventional 95% CI	P value	FDR-adjusted P value	Bias-reduced 95% CI	Bias-reduced P value	Bias-reduced FDR-adjusted P value
Training strain rolling mean	-0.176	Original scale	-0.176	-9.889×10^{-6}	-3.920×10^{-5} to 1.943×10^{-5}	0.509	0.933	-4.904×10^{-5} to 2.926×10^{-5}	0.621	0.964
Training strain rolling CV	0.637	Original scale	0.637	0.156	-0.241 to 0.553	0.440	0.933	-0.343 to 0.656	0.540	0.964
Training monotony rolling mean	-0.688	Original scale	-0.688	-0.381	-1.069 to 0.306	0.277	0.933	-1.310 to 0.547	0.421	0.964
Training monotony rolling CV	0.503	Original scale	0.503	0.019	-0.659 to 0.697	0.956	0.956	-0.811 to 0.849	0.964	0.964
Testosterone rolling mean	0.107	Original scale	0.107	1.651×10^{-4}	-3.846×10^{-4} to 7.149×10^{-4}	0.556	0.933	-5.579×10^{-4} to 8.882×10^{-4}	0.654	0.964
Testosterone rolling CV	2.086	log10	-0.342	-0.061	-0.681 to 0.558	0.846	0.933	-0.974 to 0.851	0.895	0.964
CK rolling mean	0.967	log10	0.214	-0.354	-1.171 to 0.463	0.396	0.933	-1.397 to 0.690	0.506	0.964
CK rolling CV	2.970	log10	0.485	0.539	0.196 to 0.882	0.002	0.025	0.063 to 1.015	0.027	0.319
Perceived sleep quality rolling mean	-0.023	Original scale	-0.023	-0.036	-0.426 to 0.354	0.855	0.933	-0.552 to 0.479	0.890	0.964
Perceived sleep quality rolling CV	2.958	log10	-0.388	0.056	-0.259 to 0.371	0.728	0.933	-0.378 to 0.490	0.801	0.964
Soreness rolling mean	1.278	log10	0.816	0.262	-2.381 to 2.905	0.846	0.933	-4.075 to 4.599	0.906	0.964
Soreness rolling CV	0.401	Original scale	0.401	0.207	-1.818 to 2.232	0.841	0.933	-2.483 to 2.897	0.880	0.964

Note. Markedly right-skewed predictors were log10-transformed; other predictors remained on the original scale.

Supplementary Table S13. Log10-scale sensitivity analysis for the six-predictor main quantile-regression model.

τ	Predictor	Original β	Log10-sensitivity β	Direction
0.25	Training strain rolling mean	5.121×10^{-5}	5.400×10^{-5}	Same direction
0.25	Training monotony rolling mean	-1.351	-1.483	Same direction
0.25	Testosterone rolling mean	4.386×10^{-4}	4.679×10^{-4}	Same direction
0.25	CK rolling mean	-7.243×10^{-4}	-0.660	Same direction
0.25	Perceived sleep quality rolling CV	1.748	0.055	Same direction
0.25	Soreness rolling mean	-0.162	-1.339	Same direction
0.5	Training strain rolling mean	2.861×10^{-5}	4.596×10^{-5}	Same direction
0.5	Training monotony rolling mean	-1.516	-2.074	Same direction
0.5	Testosterone rolling mean	4.427×10^{-4}	4.631×10^{-4}	Same direction
0.5	CK rolling mean	-7.823×10^{-4}	-0.639	Same direction
0.5	Perceived sleep quality rolling CV	1.972	-0.135	Opposite direction
0.5	Soreness rolling mean	0.152	0.394	Same direction
0.75	Training strain rolling mean	1.203×10^{-5}	1.328×10^{-5}	Same direction
0.75	Training monotony rolling mean	-0.886	-1.212	Same direction
0.75	Testosterone rolling mean	6.039×10^{-4}	7.955×10^{-4}	Same direction
0.75	CK rolling mean	-0.001	-0.715	Same direction
0.75	Perceived sleep quality rolling CV	1.587	0.191	Same direction
0.75	Soreness rolling mean	0.227	2.057	Same direction

Note. Direction indicates whether the coefficient retained the same sign as in the original-scale main model.

Supplementary Table S14. Convergence status for the continuous-quantile and sensitivity analyses.

Model	τ	Convergence status
Six-predictor main model (original scale)	0.1	Converged
Six-predictor main model (original scale)	0.15	Converged
Six-predictor main model (original scale)	0.2	Converged
Six-predictor main model (original scale)	0.25	Converged
Six-predictor main model (original scale)	0.3	Converged
Six-predictor main model (original scale)	0.35	Convergence limitation
Six-predictor main model (original scale)	0.4	Converged
Six-predictor main model (original scale)	0.45	Converged
Six-predictor main model (original scale)	0.5	Converged
Six-predictor main model (original scale)	0.55	Converged
Six-predictor main model (original scale)	0.6	Converged
Six-predictor main model (original scale)	0.65	Converged
Six-predictor main model (original scale)	0.7	Converged
Six-predictor main model (original scale)	0.75	Converged
Six-predictor main model (original scale)	0.8	Converged
Six-predictor main model (original scale)	0.85	Converged
Six-predictor main model (original scale)	0.9	Converged
Six-predictor main model (log10 sensitivity)	0.25	Converged
Six-predictor main model (log10 sensitivity)	0.5	Converged
Six-predictor main model (log10 sensitivity)	0.75	Converged
12-feature extended model (original scale)	0.25	Converged
12-feature extended model (original scale)	0.5	Convergence limitation
12-feature extended model (original scale)	0.75	Converged

Note. Convergence status is reported for the analyses described in the manuscript.
